# Pubertal high fat diet: effects on mammary cancer development

**DOI:** 10.1186/bcr3561

**Published:** 2013-10-25

**Authors:** Yong Zhao, Ying Siow Tan, Mark D Aupperlee, Ingeborg M Langohr, Erin L Kirk, Melissa A Troester, Richard C Schwartz, Sandra Z Haslam

**Affiliations:** 1 Breast Cancer and the Environment Research Program, Department of Physiology, Michigan State University, East Lansing, MI 48824, USA; 2 Department of Microbiology and Molecular Genetics, Breast Cancer and the Environment Research Program, Michigan State University, East Lansing, MI 48824, USA; 3 Department of Pathobiology and Diagnostic Investigation, Michigan State University, East Lansing, MI, USA; 4 Department of Epidemiology, Lineberger Comprehensive Cancer Center, University of North Carolina at Chapel Hill, Chapel Hill, NC, USA; 5 Department of Pathology and Laboratory Medicine, Lineberger Comprehensive Cancer Center, University of North Carolina at Chapel Hill, Chapel Hill, NC, USA; 6 Department of Physiology, Biomedical and Physical Sciences Building, Room 2201, 567 Wilson Road, Michigan State University, East Lansing, MI 48824, USA

## Abstract

**Introduction:**

Epidemiological studies linking dietary fat intake and obesity to breast cancer risk have produced inconsistent results. This may be due to the difficulty of dissociating fat intake from obesity, and/or the lack of defined periods of exposure in these studies. The pubertal mammary gland is highly sensitive to cancer-causing agents. We assessed how high fat diet (HFD) affects inflammation, proliferative, and developmental events in the pubertal gland, since dysregulation of these can promote mammary tumorigenesis. To test the effect of HFD initiated during puberty on tumorigenesis, we utilized BALB/c mice, for which HFD neither induces obesity nor metabolic syndrome, allowing dissociation of HFD effects from other conditions associated with HFD.

**Methods:**

Pubertal BALB/c mice were fed a low fat diet (12% kcal fat) or a HFD (60% kcal fat), and subjected to carcinogen 7,12-dimethylbenz[a]anthracene (DMBA)-induced tumorigenesis.

**Results:**

HFD elevated mammary gland expression of inflammatory and growth factor genes at 3 and 4 weeks of diet. Receptor activator of nuclear factor kappa-B ligand (RANKL), robustly induced at 4 weeks, has direct mitogenic activity in mammary epithelial cells and, as a potent inducer of NF-κB activity, may induce inflammatory genes. Three weeks of HFD induced a transient influx of eosinophils into the mammary gland, consistent with elevated inflammatory factors. At 10 weeks, prior to the appearance of palpable tumors, there were increased numbers of abnormal mammary epithelial lesions, enhanced cellular proliferation, increased growth factors, chemokines associated with immune-suppressive regulatory T cells, increased vascularization, and elevated M2 macrophages. HFD dramatically reduced tumor latency. Early developing tumors were more proliferative and were associated with increased levels of tumor-related growth factors, including increased plasma levels of HGF in tumor-bearing animals. Early HFD tumors also had increased vascularization, and more intra-tumor and stromal M2 macrophages.

**Conclusions:**

Taken together in this non-obesogenic context, HFD promotion of inflammatory processes, as well as local and systemically increased growth factor expression, are likely responsible for the enhanced tumorigenesis. It is noteworthy that although DMBA mutagenesis is virtually random in its targeting of genes in tumorigenesis, the short latency tumors arising in animals on HFD showed a unique gene expression profile, highlighting the potent overarching influence of HFD.

## Introduction

Eradication of breast cancer will be significantly advanced by the development of effective prevention strategies. Dietary fat intake and increased body mass index (BMI)/obesity have been studied for their potential contributions to breast cancer risk. High BMI (BMI ≥25 kg/m^2^) is a recognized risk factor for postmenopausal breast cancer in the pooled analysis of data from large, prospective cohort studies [1]. Conversely, in the same analysis, high BMI (BMI >31) is associated with reduced risk for premenopausal breast cancer [1]. Similarly, weight gain in adult years is associated with increased risk for postmenopausal breast cancer and reduced risk for premenopausal breast cancer [2]. The relationship between dietary factors, specifically dietary fat, the major contributor to increased BMI, and breast cancer risk is less clear. Recent research has demonstrated no associations with breast cancer risk for adult intake of total fat, saturated fat, or other specific types of dietary fat. These findings did not vary by ethnicity, estrogen/progesterone receptor status, tumor stage, BMI, hormone replacement therapy use, follow-up period, family history of breast cancer, or smoking status at baseline [3]. Lack of associations between dietary factors and breast cancer risk could be the result of numerous sources of bias, including misclassification of dietary intake. Furthermore, the time period in which diet may play the most important role is unclear. In this regard, dietary data usually reflect diet for the year prior to diagnosis or in adulthood prior to breast cancer. Thus, there is a need for a better understanding of the relative contributions of diet, and the timing of diet and/or obesity, to breast cancer risk.

Based on studies in humans and rodents, there is now wide recognition that the origins of breast cancer probably occur early in development, especially during the times of rapid breast development in the pubertal transition [4]. Emerging evidence indicates that the composition, cellular proliferation, and maturation of the gland can be altered by diet and environmental exposures, and that exposure during puberty is particularly relevant [5]. An important gap in our understanding is how diet and/or increased BMI specifically influence pubertal breast development and breast cancer risk in adulthood. The typical western diet, high in saturated fat, is largely credited for the obesity epidemic in the US. However, it should be noted that there are more people who eat a high-fat western diet and potentially suffer its consequences, than are actually obese. At the same time, the effects of diet versus those of increased BMI are difficult to distinguish, since a high fat diet (HFD) often results in increased BMI.

Among the mechanisms proposed for diet/obesity-associated breast cancer risk are altered glucose metabolism, altered steroid hormone levels, and inflammatory processes [6]. It is entirely possible that HFD during puberty may alter breast development, independently of increasing BMI, through one or more of these mechanisms, thereby modifying the risk for breast cancer.

Ovarian hormones and growth factors are primary factors driving pubertal mammary gland development in humans and rodents. Estrogen (E) and progesterone (P) promote epithelial cell proliferation by inducing amphiregulin (Areg), a growth factor produced in estrogen receptor α (ERα)- and progesterone receptor (PR)-positive cells, that acts through a paracrine mechanism in the stroma and in ER negative mammary epithelial cells [7,8].

Macrophages and eosinophils play important roles in normal pubertal mammary gland development in the mouse [9] and, in the case of macrophages, also contribute to mammary tumor progression [10]. Macrophage association with terminal end buds is needed for ductal elongation and eosinophils are required for proper ductal development, particularly branching. Mast cells have also been implicated in pubertal mammary gland ductal morphogenesis, with a role independent of that of macrophages [11]. Importantly, inflammatory leukocytes are involved in several animal models of mammary tumor progression [12,13]. A diet high in saturated fat, that may increase inflammatory processes in the mammary gland, may also promote mammary tumorigenesis [14-17].

We previously investigated the impact of HFD on pubertal mammary gland development in BALB/c mice [18]. Pubertal mice were fed non-isocaloric diets that were either high in saturated fat (HFD, 60% kcal from fat) or low in saturated fat (LFD, 12% kcal from fat) during the peri-pubertal period from 3 to 7 weeks of age. Notably, HFD significantly increased mammary epithelial cell proliferation without a significant increase in body weight [18]. That study showed a small increase in fasting blood glucose and plasma insulin levels, and no difference in plasma E levels after 4 weeks on the diets.

The purpose of the present study was to investigate the effects of HFD initiated in the peri-pubertal period on mammary gland susceptibility to DMBA-induced mammary tumorigenesis in adulthood in a non-obesogenic context in BALB/c mice. We sought to examine the potential effects on proliferation, inflammatory processes, glucose metabolism and altered hormone levels in the context of mammary tumorigenesis.

The results obtained showed a significant decrease in tumor latency in HFD-fed mice. The association of HFD-induced decreased latency with local and systemically increased growth factor expression, and promotion of inflammatory and angiogenic processes, suggest likely mechanisms for enhanced tumorigenicity. Importantly, these HFD-induced effects occurred without significant body weight gain, obesity, or major effects on glucose and insulin levels, or E and P levels. Furthermore, tumors occurring with reduced latency on HFD displayed a gene expression profile that clearly distinguished them from tumors occurring on LFD.

## Materials and methods

### Animals

Three-week-old female BALB/c mice were obtained from Charles River Laboratories (Portage, MI, USA). Mothers of these mice were maintained on LabDiet 5 L79 (PMI Nutrition International LLC, St. Louis, MO, USA) before and during pregnancy, and while nursing. Upon arrival, mice were randomly distributed into two non-isocaloric diet groups, LFD or HFD. Animals were housed in polysulfone cages, and received measured amounts of food and water *ad libitum*. Housing facilities were maintained on a 12:12 h light–dark cycle, at 20 to 24°C with 40 to 50% relative humidity. All animal experimentation was conducted in accord with accepted standards of humane animal care and approved by the All University Committee on Animal Use and Care at Michigan State University.

### Diets

Diets were initiated at 3 weeks of age and maintained throughout the experimental period up to 45 weeks of age. Two diets (I and II) were used in these studies. Diet I: low fat diet 58G7 (12% kcal fat - LFD) and high fat diet 58G9 (60% kcal fat - HFD) were purchased from TestDiet (PMI Nutrition International LLC). Initial concern over the carbohydrate content of Diet I and the potential for the development of metabolic syndrome led us to also use Diet II, which contained maltodextrin instead of the dextrin and sucrose contained in Diet I. Diet II: low fat diet 12450B (10% kcal fat - LFD) and high fat diet 12492 (60% kcal fat - HFD), both with corn oil replacing soy oil, were purchased from Research Diets (New Brunswick, NJ, USA). For Diet I, 11% kcal fat came from corn oil, with the remainder of the fat (1 or 49% kcal) coming from lard. For Diet II, 5.5% kcal fat came from corn oil, with the remainder of the fat (4.5 or 54.5% kcal) coming from lard. Detailed composition of Diets I and II is described in Additional file 1. LFD and HFD Diets I and II did not differ significantly with regard to their effects on body weight, blood glucose, insulin levels, tumor incidence, or tumor latencies (see Additional files 2, 3, 4, 5). Therefore, results obtained on tumorigenesis with the two diets were combined.

### Tumorigenesis

Mice were treated with 7,12-dimethylbenz(a)anthracene (DMBA) (Sigma-Aldrich, St. Louis, MO, USA) prepared in vegetable oil and administered by oral gavage (50 mg/kg body weight/mouse) once per week for 4 weeks starting at 5 weeks of age. Body weights were monitored weekly, and animals were palpated for tumors once a week starting at 8 weeks post first DMBA dose (see Additional file 6). Tumor volume was measured twice per week, and harvested at 1 cm size. At 2 h prior to sacrifice, mice were injected with 5-bromo-2’-deoxyuridine (BrdU) (70 μg/g body weight; Sigma-Aldrich) for analysis of cellular proliferation. At termination of all feeding studies, portions of tumors and mammary tissues were either snap frozen for protein and RNA isolation, or formalin fixed and either processed as whole mounts [19] or paraffin embedded for H&E staining and immunohistochemistry [20]. Whole-mount preparations of glands and H&E sections were scored for the presence of hyperplasia and neoplasia [21]. There were no notable diet effects on mammary gland morphology as assessed by whole mounts or histological sections at the time points analyzed. All lesions and tumors were reviewed and classified, as previously described [22].

### Metabolic parameters

Plasma glucose and insulin levels were the metabolic parameters measured. Non-fasting, randomly sampled glucose and insulin levels were obtained from mice fed ad libitum, as an appropriate and acceptable method based on mouse feeding habits and the stress caused by fasting [23]. Plasma levels of glucose were determined by AccuChek Compact Glucometer (Roche, Nutley, NJ, USA) and insulin levels were determined with an insulin ELISA kit from EMD Millipore (Cat. number: EZRMI-13 K; St. Charles, MO, USA), following the manufacturer’s instructions.

### Estrogen and progesterone assay

Total serum E levels were determined using Delfia Estradiol time-resolved fluoroimmunoassay (Cat. number: 1244–056; PerkinElmer, Turku, Finland), following the manufacturer’s instructions. The levels of serum P were measured by ELISA (Cat. number: 11-PROHU-E01; Alpco Diagnostics, Salem, NH, USA), following the manufacturer's instructions.

### Estrogen receptor analysis

Mammary gland or tumor sections (5 μm) were prepared and subjected to antigen retrieval and immunofluorescent staining, as previously described [20]. Briefly, sections were blocked with goat anti-mouse immunoglobulin G (IgG) Fab (antigen binding fragment; 1:100 with 1% BSA in PBS (PBSA)) for 1 h at room temperature (RT), followed by blocking with normal goat serum in PBS for 30 minutes at RT. Then, sections were incubated with mouse anti-ERα (1:10 in PBS-0.5% Triton X-100; Cat. number: NCL-ER-6 F11; Novocastra Laboratories Ltd, Newcastle upon Tyne, UK) at 4°C overnight. After a brief wash, sections were incubated with an Alexa 488-labeled goat anti-mouse secondary antibody (Ab) (1:200 in PBS; Invitrogen Molecular Probes, Grand Island, NY, USA) at RT for 30 minutes, and then counterstained with 4’,6-diamidino-2-phenylindole (DAPI) for 5 minutes. The stained sections were visualized with a Nikon Eclipse TE2000-U fluorescence microscope (Nikon, Inc., Melville, NY, USA) using a 40X objective lens, and the captured fluorescent images were analyzed using MetaMorph software (Molecular Devices, LLC, Sunnyvale, CA, USA). A minimum of 1,000 cells were counted for each tumor. Tumors were considered to be ERα-positive (ER+) if 10% or more of the total cells counted were ER + [24].

### Macrophage analysis

Mammary gland or tumor sections (5 μm) were prepared for immunofluorescent staining [20]. Briefly, after deparaffinization, mammary gland sections were subjected to antigen retrieval at 121°C and 15 pounds per square inch (psi) for 5 minutes; tumor sections did not receive antigen retrieval, as the integrity of tumor sections was compromised by the procedure, and staining was adequate in its absence. All sections were treated with proteinase K (20 μg/mL in Tris-EDTA buffer, pH 8.0) at 37°C for 3.5 minutes. After brief washes with PBS, sections were blocked with normal rabbit serum in PBS, followed by incubation with goat anti-Arginase 1 (Arg1) (1:200 in PBS-0.5% Triton X-100; Cat. number: sc-18354; Santa Cruz Biotechnology, Inc., Santa Cruz, CA, USA) at 4°C overnight. After brief washes, sections were incubated with Alexa 546-labeled rabbit anti-goat secondary Ab (1:100 in PBS; Cat. number: A21085; Invitrogen Molecular Probes) at RT for 30 minutes and then blocked with normal goat serum in PBS for 30 minutes. The sections were then incubated with rat monoclonal anti-F4/80 (1:75 in PBS-0.5% Triton X-100; Cat. number: MCA497R; AbD Serotec, Raleigh, NC, USA) at 4°C overnight, followed by incubation with Alexa 488-labeled goat anti-rat secondary Ab (1:100 in PBS; Invitrogen Molecular Probes) at RT for 30 minutes and counterstaining with DAPI for 5 minutes. The stained sections were visualized with a Nikon Eclipse TE2000-U fluorescence microscope (Nikon, Inc.) using a 40X objective lens, and the captured fluorescent images were analyzed using MetaMorph software (Molecular Devices, LLC). The number of F4/80 and/or Arg1-positive cells is expressed as cells per structure in the mammary gland peri-epithelial area, and cells per image in tumor samples.

### Cellular proliferation analysis

Mammary gland or tumor sections (5 μm) were prepared and immunoperoxidase staining was performed [20]. Briefly, after deparaffinization, sections were incubated in 3% of hydrogen peroxide (H_2_O_2_) in methanol for 10 minutes. Then sections were subjected to antigen retrieval at 121°C and 15 psi for 15 minutes. Sections were blocked with goat anti-mouse IgG Fab (1:100 in 1% PBSA) for 1 h, followed by blocking with normal goat serum in PBS. Then, sections were incubated with mouse anti-BrdU (1:100 in PBS-0.5% Triton X-100; Cat. number: ab27958; Abcam plc, Cambridge, MA, USA) at 4°C overnight. After a brief wash, sections were incubated with goat anti-mouse biotin secondary Ab (1:400 in PBS; DAKO Denmark A/S, Glostrup, Denmark) at RT for 30 minutes, and then incubated with ABC reagent (PK-7100, Vector laboratories, Inc., Burlingame CA, USA) for 30 minutes. The sections were then incubated with metal-enhanced 3,3-diaminobenzidine (DAB) substrate solution (100 μL of DAB substrate + 900 μL stable peroxide substrate buffer; Thermo Scientific, Rockford, IL, USA) for 7 minutes and counterstained with hematoxylin for 2 minutes. The stained sections were visualized with a Nikon Eclipse E400 light microscope (Nikon, Inc.) using a 40X objective lens. A minimum of 1,000 cells were counted for each section, and a minimum of two to three tissue sections per animal were analyzed. The number of BrdU-positive cells is expressed as the percentage of total epithelial cells counted.

### Immunohistochemical analysis of blood vessel density

Mammary gland or tumor sections (5 μm) were deparaffinized and were incubated in 2% H_2_O_2_ in methanol/PBS (1:1 ratio) for 30 minutes followed by antigen retrieval by boiling at 95°C for 5 minutes. Sections were then treated for 2 h with rabbit anti-CD31 (1:50 in PBS-0.5% Triton X-100; Cat. number: AP15436PU-N; Acris Antibodies, Inc., San Diego, CA, USA) for endothelial cell staining and detection of blood vessels. After a brief wash, sections were incubated with secondary swine anti-rabbit Ab at RT for 30 minutes, and then incubated with ABC reagent (PK-7100, Vector laboratories, Inc.) for 30 minutes. The sections were then incubated with metal-enhanced DAB substrate solution (100 μL of DAB substrate + 900 μL stable peroxide substrate buffer; Thermo Scientific) for 7 minutes and counterstained with hematoxylin for 2 minutes. The stained sections were visualized with a Nikon Eclipse E400 light microscope (Nikon, Inc.) using a 40X objective lens. A minimum of 1,000 cells were counted for each section, and a minimum of two to three tissue sections per animal were analyzed. Digital micrographs were captured and the images were overlaid with grids containing 240 squares (324 μm^2^/square). Blood vessel density is expressed as the percentage of CD31-positive squares.

### Eosinophil and mast cell analysis

Deparaffinized 5-μm sections were stained with Astra Blue/Vital New Red [25]. Sections dehydrated in 95% ethanol for 5 sec were incubated in Astra Blue solution (5 mg/mL in 75% ethanol) for 30 minutes at RT. After rinsing in double-distilled water (H_2_O), sections were again dehydrated in 95% ethanol for 5 sec, and then incubated in Vital New Red solution (0.2 g/L in 50% ethanol) for 1 h at RT and counterstained with hematoxylin for 2 minutes. The stained sections were visualized with a Nikon Eclipse E400 light microscope (Nikon, Inc.) using a 40X objective lens. The number of eosinophils and mast cells is expressed as cells per structure in the mammary gland peri-epithelial area.

### Mammary gland structure analysis

Sections stained for macrophages, cellular proliferation, eosinophils, and mast cells were analyzed by mammary gland epithelial structure: small ducts, large ducts, terminal end buds (TEBs), or hyperplastic foci. Large ducts were qualitatively characterized by larger lumen diameter, comprising greater than 50 cells in a section, and by an extensive extracellular matrix and fibroblasts surrounding the epithelium. Conversely, small ducts were qualitatively characterized by smaller lumen diameter, comprising fewer than 50 cells, and by a limited extracellular matrix surrounding the epithelium. TEBs were characterized by location within the gland, presence of multiple epithelial cell layers, and direct apposition of epithelium to adipocytes without extracellular matrix. Hyperplastic structures had multiple cell layers of noticeably distorted epithelium compared to normal epithelial structures.

### Quantitative PCR arrays and quantitative reverse transcription-PCR analysis (qRT-PCR)

Total RNA was isolated from mouse mammary glands (intact or cleared fat pads) or tumors using TRIzol® reagent (Invitrogen, Carlsbad, CA, USA) and purified using the RT^2^ qPCR-Grade RNA isolation kit (SABiosciences, Frederick, MD, USA). We used 3 μg of total RNA for first strand cDNA synthesis using the RT^2^ First Strand kit (SABiosciences), following the manufacturer’s instructions. The cDNA (20 μL) were diluted to 150 μL with de-ionized H_2_O. RNA expression was then analyzed using targeted PCR arrays (SABiosciences) for Breast Cancer (PAMM-131), Cancer Pathway Finder (PAMM-033), Growth Factors (PAMM-041), and Inflammatory Cytokines and Receptors(PAMM-011). In addition to the arrays, primers for the following selected RNA were purchased from SABiosciences: *Tnfs11* (*RANKL*) (PPM03047E), *18S ribosomal RNA* (*rRNA*) (PPM57735E), *Ribosomal protein L32* (*RPL32*) (PPM03300B). For qRT-PCR analysis, each reaction (25 μL) included 12.5 μL of 2X SABiosciences RT^2^ qPCR Master Mix (SYBR Green), 1 μL of diluted first-strand cDNA synthesis reaction, and 11.5 μL of de-ionized H_2_O. qRT-PCR was performed with the ABI 7500 Fast Real-Time PCR System (Applied Biosystems, Inc., Foster City, CA, USA) using the following program: step 1: 95°C, 10 minutes; step 2: 40 cycles of 95°C, 15 s and 60°C, 1 minute; step 3: dissociation curve 95°C, 1 minute; 65°C, 2 minutes (optics off); 65 to 95°C at 2°C per minute (optics on). The data were analyzed using online software from SABiosciences [26]. For *RANKL* RNA, the comparative cycle threshold (CT) method was used to calculate the fold change in gene expression after normalization to the values for *18S rRNA* and *RPL32* RNAs. RNA from the number 4-inguinal mammary glands of three animals was analyzed from each treatment group. In the tumor analysis, the three HFD-E tumors were adenosquamous (ER-), ductal (ER+) and cribriform (ER-) adenocarcinomas with tumor onset at 10, 14 and 17 weeks post-DMBA, respectively. The three LFD tumors were adenosquamous (ER-), papillary (ER+) and cribriform (ER-) adenocarcinomas with tumor onset at 31, 34 and 38 weeks post-DMBA, respectively.

### Pathway analysis

Genes that were significant (unadjusted *p*-value <0.05) in univariate analyses were evaluated for ontological enrichment using Ingenuity Pathway Analysis (IPA, Ingenuity® Systems, Redwood City, CA, USA), with Benjamini-Hochberg (B-H) multiple testing correction. The background was set to include only the genes on the qPCR arrays analyzed for each sample group. Significant functions and pathways were defined as those with at least two significant genes per pathway and with B-H *P*-values less than 0.05.

### Analysis of plasma cytokine levels

Plasma levels of 20 different cytokine proteins (AREG, basic fibroblast growth factor (bFGF), epidermal growth factor (EGF), hepatocyte growth factor (HGF), insulin-like growth factor 2 (IGF-2), IL-6, leptin, receptor activator of nuclear factor kappa-B ligand (RANKL), vascular endothelial growth factor (VEGF), resistin, TNFα, IL-1α, IL-1β, insulin-like growth factor binding protein (IGFBP)-2, IGFBP-3, prolactin, T-cell activation (TCA)-3 (CCL1), macrophage colony stimulating factor (M-CSF), epiregulin, and osteoprotegerin (OPG)) were determined by cytokine Ab arrays (Cat. number: AAM-CUS-G; RayBiotech, Norcross, GA, USA), following the manufacturer’s instructions. Briefly, the glass array slide was incubated with blocking buffer for 30 minutes at RT, and then incubated with diluted mouse plasma (30 μL plasma diluted to 120 μL with blocking buffer) overnight at 4°C with slow shaking. Then, after thorough washing with washing buffer, the array slide was incubated with biotin-labeled Ab for 2 h at RT with slow shaking. After thorough washing with washing buffer, the array slide was incubated with HiLytePlus™ 555 Fluor-conjugated streptavidin for 2 h at RT with slow shaking. Then, after thorough washing, the slide was dried and absolute fluorescent intensity measured by an Agilent G2505B laser scanner (Agilent Technologies, Inc., Santa Clara, CA, USA). The data were analyzed by software provided by the company. Additionally, plasma IGF-1 levels were measured using a mouse IGF-1 ELISA kit (ELM-IGFI-001; RayBiotech), according to the manufacturer’s directions.

### Statistical analyses

The PCR arrays were statistically analyzed using proprietary software from SABiosciences. Correlations between cytokine mRNA levels and leukocyte populations were determined by Spearman’s method [27]. Otherwise, results are expressed as mean ± standard error of the mean (SEM). Differences were considered significant at *P* <0.05 using the Student *t*-test or analysis of variance (ANOVA) followed by the Tukey multiple comparison test, as appropriate. Tumor incidence was analyzed by the Chi-square test. Tumor latencies were determined from Kaplan-Meier plots.

## Results

### HFD decreases tumor latency

**Figure 1:**
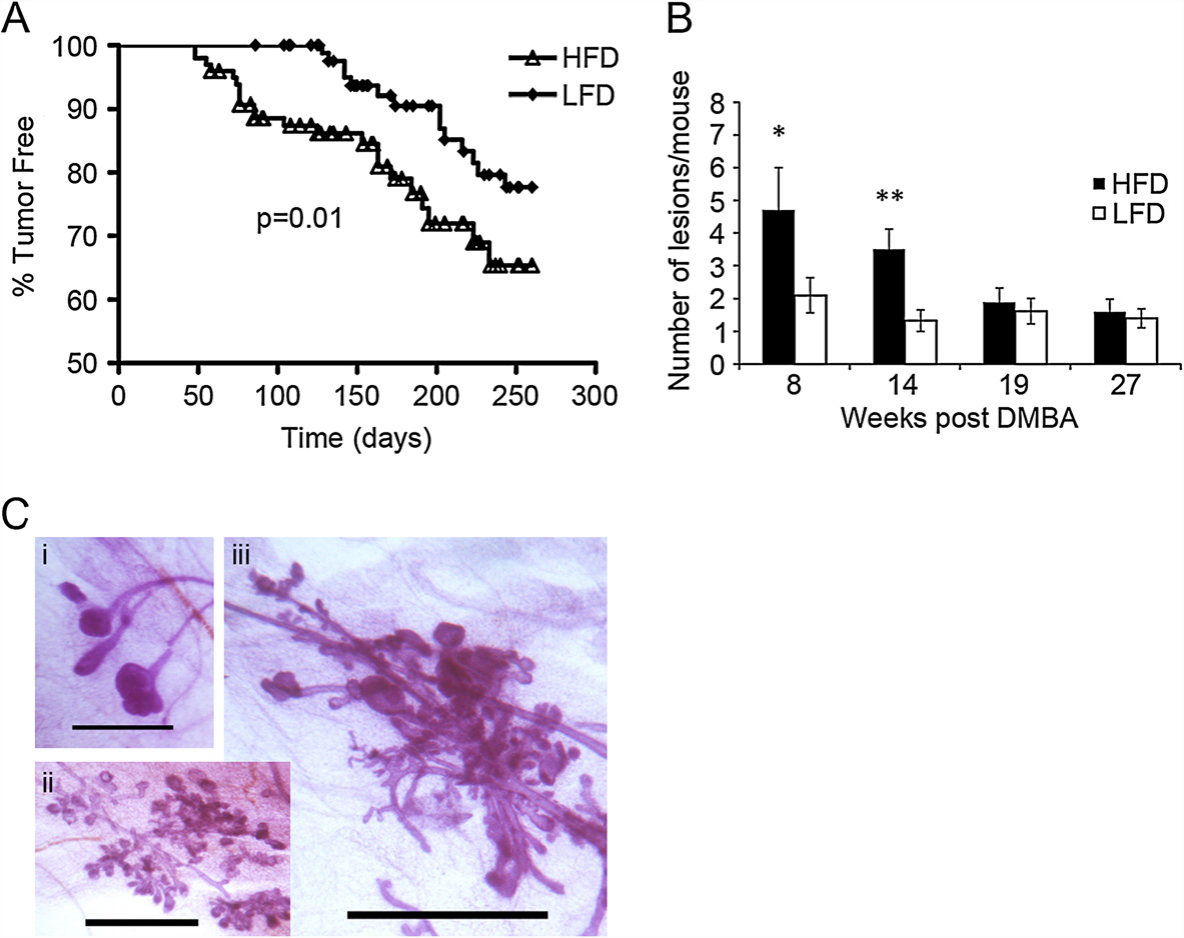
**Characteristics of tumor development in high fat diet- versus low fat diet-fed mice. (A)** Kaplan-Meier plot of all tumors developing in high fat diet (HFD)- and low fat diet (LFD)-fed mice. Time = number of days post last 7,12-dimethylbenz[a]anthracene (DMBA) treatment (HFD mice, n = 95; LFD mice, n = 87) **(B)** Time-course of epithelial proliferative lesion development. More hyperplastic and precancerous lesions developed in HFD-fed DMBA-treated mice at 8 and 14 weeks post first DMBA treatment. Bars represent mean ± standard error of the mean of lesions per mouse; n = 5 mice at each time for HFD and LFD. ^*^P = 0.05; ^**^P = 0.003. **(C)** Epithelial proliferative lesions comprised (i) terminal duct hyperplasia, (ii) lobular hyperplasia, and (iii) mixed dysplasia. Scale bar = 1 mm.

A Kaplan-Meier plot (Figure 1A) shows that HFD-fed mice developed tumors significantly earlier than LFD-fed mice (*P* = 0.01) with a significantly reduced median time to tumor onset (204 days LFD versus 115 days HFD; *P* = 0.00075). Although HFD caused a 1.8-fold increase in tumor incidence (LFD, 14.95%, n = 87 mice versus HFD, 26.3%, n = 95 mice; *P* = 0.059), this did not reach statistical significance. Notably, there were no significant differences in body weight (see Additional file 2) or in parametrial fat pad weights (data not shown) between animals fed HFD and LFD over the course of the tumor study.

Since decreased latency was the major difference between the two diets, we focused on investigating the basis for the latency difference. To that end, we focused our analysis on comparing early developing tumors on HFD (HFD-E) tumors with LFD tumors. Representative HFD-E tumors were selected from tumors that developed before the earliest observed LFD tumors, at 19 weeks or less post-first DMBA treatment. Conversely, representative LFD tumors were selected after 19 weeks or more post-first DMBA treatment, when LFD tumors were first detected. We considered the possibility that reduced latency in HFD-fed mice might be due to accelerated development and increased numbers of atypical hyperplastic, pre-cancerous lesions. Time-course analysis showed that, indeed, HFD-fed mice developed significantly more hyperplastic lesions per mouse after 8 and 14 weeks post-first DMBA treatment (10 and 16 weeks on diet, respectively), times before palpable tumors were detected (Figure 1B). The hyperplastic lesions comprised ductal and alveolar hyperplasia for both diets (Figure 1C). There were no significant differences in tumor types or ER status (Table 1).

**Table 1:** Tumor histopathology

**Tumor**	**N**	**Adenocarcinoma type**	**ER status**^**a**^
Low fat diet	13	7/13 glandular/acinar	7/13 ER+
		3/13 adenosquamous	
		3/13 papillary	
High fat diet-early	10	3/10 glandular/acinar	6/10 ER+
		7/10 adenosquamous	

^a^Estrogen receptor (ER) status based on >10% receptor-positive cells. High fat diet-early represents mice with early developing tumors while on the high fat diet.

### Effects of diet on mammary gland and tumor proliferation

**Figure 2:**
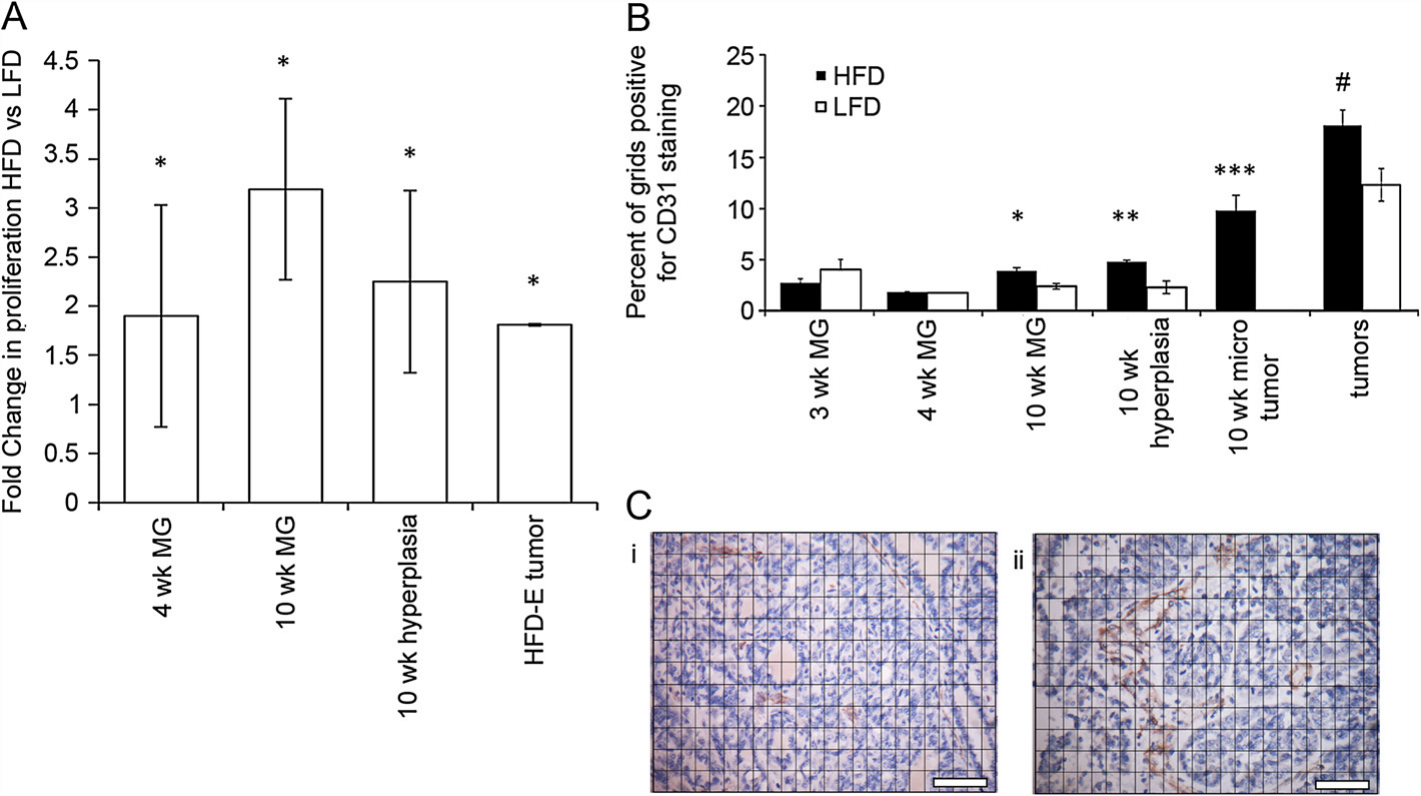
**Proliferation and angiogenesis in mammary glands, tumors, and tumor microenvironments in mice fed on high fat and low fat diets. (A)** Proliferation: fold increases in proliferation in normal mammary epithelium at 4 and 10 weeks, hyperplastic foci at 10 weeks, and in tumor epithelium from mice on a high fat diet (HFD) versus low fat diet (LFD). At 4 weeks, mice fed HFD exhibited increased cellular proliferation as measured by proliferating cell nuclear antigen (PCNA); ^*^P <0.05. Note, the 4-week data are a re-analysis of data published in Olson *et al*. (2008). At 10 weeks, mice fed HFD exhibited increased cellular proliferation in both normal epithelium and hyperplastic foci, as measured by 5-bromo-2’-deoxyuridine (BrdU) incorporation; ^*^P <0.05. Early developing tumors on HFD (HFD-E) also exhibited increased cellular proliferation, as measured BrdU incorporation; ^*^P <0.05. **(B)** Angiogenesis: blood vessel density was measured, as described in Materials and Methods, by the area occupied by CD31-positive vessels near normal mammary epithelium at 3, 4, and 10 weeks, and in tumor epithelium from mice on HFD versus LFD. At 10 weeks, CD31-stained vessels were significantly increased adjacent to normal mammary gland structures (^*^P = 0.01), hyperplastic foci (^**^P = 0.04) and microscopic tumors (tumors versus hyperplasia; ^***^P = 0.02) in HFD-fed compared to low fat diet (LFD)-fed mice. CD31 staining was also greater in HFD-E tumors (^#^*P* = 0.01) compared to LFD tumors. **(C)** Insets show increased CD31 staining of (ii) an HFD-E tumor compared with (i) an LFD tumor. Scale bars = 50 mm.

We examined mammary epithelial cell and tumor cell proliferation, as determined by BrdU incorporation, at 10 weeks on diet and in tumors (Figure 2A). These measurements were compared to our earlier report of HFD-enhanced proliferation at 4 weeks on diet [18]. At both 4 and 10 weeks on diet, there were significant increases in epithelial cell proliferation in HFD-fed mice, with the highest proliferation seen at 10 weeks in normal mammary structures and hyperplastic lesions. Proliferation was also significantly greater in HFD-E tumors, but was less than that seen after 10 weeks on HFD.

Since tissues and tumors require adequate vascularization to sustain proliferation, we also measured angiogenesis, as determined by blood vessel density (Figure 2B, C). At 3 and 4 weeks on diet, blood vessel density was low and independent of diet. At 10 weeks on diet, HFD-fed mice exhibited significantly increased blood vessel density around normal mammary gland structures and foci of atypical hyperplasia compared with LFD-fed mice. Notably, blood vessel density was highest around microscopic, non-palpable tumors in HFD-fed mice. HFD-E tumors also exhibited increased blood vessel density.

To gain further insight into the enhanced proliferative characteristics observed on HFD, we analyzed RNA expression by qRT-PCR using PCR arrays targeted to Growth Factors (Table 2). At 3 weeks, *Fgf18* and *Il4* were upregulated, and at 4 weeks, *brain derived neurotrophic factor* (*Bdnf*) and *leukemia inhibitory factor* (*Lif*) were upregulated, whereas *Lefty2* was downregulated. Interestingly, at 4 weeks on HFD, only *RANKL* was robustly upregulated 17-fold. RANKL is a P-regulated paracrine growth factor in the mammary gland [28-30], and HFD-induced *RANKL* upregulation could contribute to the increased proliferation observed at 4 weeks on diet. There was no specific overlap in mammary gland gene expression at 3 or 4 weeks on diet.

**Table 2:** Targeted Q-PCR analysis of mammary glands after 3, 4, or 10 weeks and mammary tumors on HFD versus LFD

**Growth factor genes**		**Fold change HFD versus LFD**			
**Symbol**	**Description**	**3 wks MG**	**4 wks MG**	**10 wks MG**	**Tumor**
*Fgf18*	*Fibroblast growth factor 18*	2.0	-	-	-
*Il4*	*Interleukin 4*	2.0	-	-	-
*Bdnf*	*Brain derived neurotrophic factor*	-	2.4	-	-
*Lefty2*	*Left-right determination factor 2*	-	-2.5	-	-
*Lif*	*Leukemia inhibitory factor*	-	2.8	-	-
*RANKL*	*Receptor activator of nuclear factor kappa-B ligand*	-	17	-	-
*Bmp2*	*Bone morphogenetic protein 2*	-	-	2.0	-
*Bmp3*	*Bone morphogenetic protein 3*	-	-	2.4	-
*Fgf10*	*Fibroblast growth factor 10*	-	-	2.0	-
*Gdf10*	*Growth differentiation factor 10*	-	-	1.9	-
*Gdf5*	*Growth differentiation factor 5*	-	-	2.2	-
*Nodal*	*Nodal*	-	-	2.4	-
*Pgf*	*Placental growth factor*	-	-	2.5	-
*Tgfa*	*Transforming growth factor alpha*	-	-	1.9	-
*Tgfb1*	*Transforming growth factor, beta 1*	-	-	1.9	-
*Vegfa*	*Vascular endothelial growth factor A*	-	-	2.1	-
*Il1a*	*Interleukin 1 alpha*	-	-	3.0	-
*Il1b*	*Interleukin 1 beta*	-	-	2.1	-
*Il2*	*Interleukin 2*	-	-	3.0	-
*Il7*	*Interleukin 7*	-	-	3.4	-
*Cxcl12*	*Chemokine (C-X-C motif) ligand 12*	-	-	1.8	-
*Bmp7*	*Bone morphogenetic protein 7*	-	-	-	3.2
*Inha*	*Inhibin alpha*	-	-	-	2.2
*Ntf3*	*Neurotrophin 3*	-	-	-	53.4
*Bmp10*	*Bone morphogenetic protein 10*	-	-	-	-3.4

N = 3 high fat diet (HFD) mice, n = 3 low fat diet (LFD) mice, n = 3 HFD mice with early developing tumors (HFD-E), n = 3 LFD mice with tumors. *P* ≤0.05 for all genes listed.

At 10 weeks on diet, a large number of growth factor genes were upregulated in mammary glands of HFD-fed mice. The one with most obvious relevance to mammary epithelial proliferation was *TGFα*[31], a ligand for the EGF receptor (EGFR). Other upregulated genes included several encoding TGFβ superfamily growth factors [32] (that is, *Bmp2*, *Bmp3*, *growth differentiation factor* (*Gdf*) *5*, *Gdf10*, *Nodal*, and *Tgfb1*), as well as genes encoding IL-2, IL-7, FGF10, PGF, VEGF-A, Chemokine (C-X-C motif) ligand (CXCL) 12, IL-1α, and IL-1β. Interestingly, there was no specific overlap with growth factors found induced at 3 and 4 weeks on HFD.

Analysis of growth factor-related gene expression in the HFD-E tumors in comparison to LFD tumors showed upregulation of *bone morphogenic protein* (*Bmp*) *7*, and *inhibin α (Ihha)*, as well as a trend toward upregulation of *Fgf15* (*P* = 0.09). *Neurotrophin 3* (*Ntf3*) was most robustly upregulated (53-fold). *Bmp10* was downregulated. It is striking that the HFD-E tumors display a unique gene expression profile that shows no overlap with the genes identified at 3, 4, and 10 weeks on diet.

### Effect of diet on inflammatory processes

**Figure 3:**
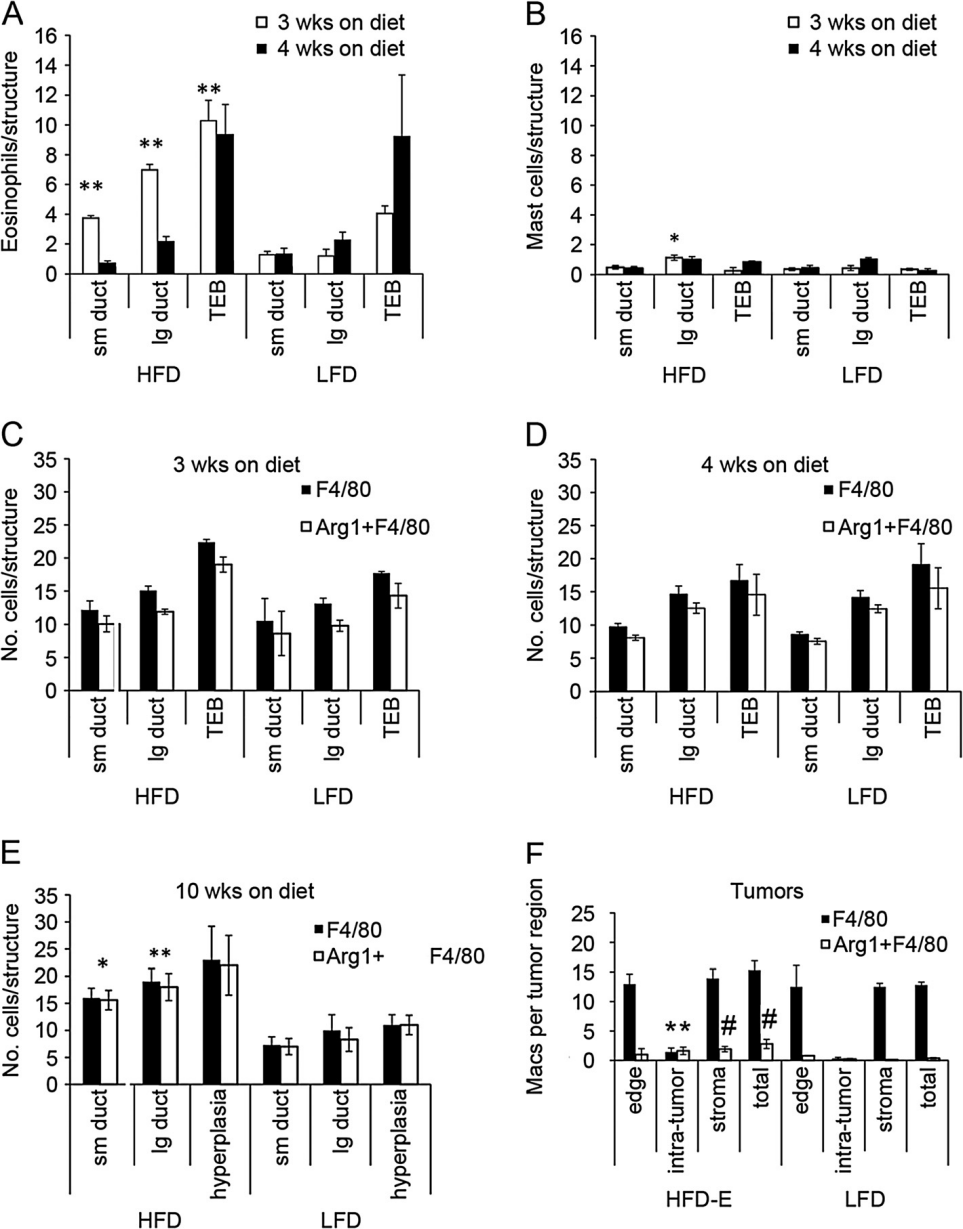
**Inflammatory cell recruitment in mammary gland, tumors, and tumor microenvironments in high fat diet- and low fat diet-fed mice. (A and B)** BALB/c mice were started on diets at 3 weeks of age and terminated after 3 or 4 weeks for analysis of eosinophil **(A)** and mast cell **(B)** recruitment to mammary gland epithelial structures, as described in the Materials and Methods. At 3 weeks on diet, eosinophil recruitment **(A)** for all mammary structures and mast cell recruitment **(B)** for large ducts was significantly increased in high fat diet (HFD) compared to low fat diet (LFD)-fed mice. ^*^P = 0.0001; ^**^P = 0.03. **(C, D, E, and F)** Sections from mice terminated at 3 weeks on diet **(C)**, 4 weeks on diet **(D)**, 10 weeks on diet **(E)** and from HFD-E and LFD tumors **(F)** were double immunofluorescently stained with anti-F4/80 and anti-Arg1 antibodies, as described in Materials and Methods, and then analyzed for macrophage recruitment. At 10 weeks, total macrophage (F4/80) and M2 macrophage (Arg1 + F4/80) recruitment **(E)** was increased adjacent to small ducts (^*^P = 0.01) and large ducts (^**^P = 0.05) in mammary glands of HFD-fed mice. The increase in F4/80 and Arg1 + F4/80 staining in HFD versus LFD hyperplasia was not significant (*P* = 0.16). Tumor-associated macrophages **(F)** were quantified based on their location at the tumor edge, within the tumor (intra-tumor), in the tumor stroma (stroma), and combined for total tumor-associated macrophages. ^*^P = 0.05 that there were more F4/80 and Arg1 + F4/80 labeled macrophages within HFD-E tumors. ^#^*P* = 0.01 that there were more Arg1 + F4/80 macrophages (total) in HFD-E tumors and within HFD-E stroma.

Leukocytes (that is, macrophages, eosinophils and mast cells) play important roles in normal pubertal mammary gland proliferation and development [9-11]. Since HFD promoted mammary epithelial cell proliferation, we also examined the effect of diet on leukocyte recruitment (Figure 3). At 3 weeks, there was a significant HFD-induced increase in recruitment of eosinophils to all mammary structures (Figure 3A) and of mast cells to large ducts (Figure 3B). At 4 weeks, this effect was no longer seen; however, high levels of eosinophils were observed around TEBs on both diets (Figure 3A). Macrophage recruitment to glandular structures was similar at 3 and 4 weeks, and was independent of diet (data not shown). The majority of macrophages were of the M2 phenotype (that is, Arg1+) for both diets.

At 10 weeks on diet, HFD-fed mice had significantly more macrophages recruited to all normal mammary gland structures (Figure 3C). With both diets, the vast majority of recruited macrophages were M2-polarized. The greater number of macrophages recruited at 10 weeks on HFD did not appear to be due to an interaction with DMBA because the same fold increases in macrophages were detected at 10 weeks on HFD diet without DMBA treatment (data not shown). There were exceedingly low numbers of eosinophils and mast cells on either diet (data not shown). There were significantly more M2 macrophages present within HFD-E tumors and within their tumor stroma (Figure 3D). There were no significant differences in the numbers of eosinophils and mast cells associated with HFD-E or LFD tumors (data not shown).

To gain a better understanding of how HFD increased leukocyte recruitment, we compared the expression of inflammation-related genes in LFD and HFD mammary glands by qRT-PCR using the Inflammatory Cytokines and Receptors PCR array (Table 3). There was no overlap in inflammation-related mRNA expressed at 3, 4, and 10 weeks on diet. Genes encoding CCL24 (*eotaxin-2*), CCL3 (*MIP-1α*), IL-4, and IL5Rα were transiently induced at 3 weeks. *RANKL* was the only upregulated inflammatory gene observed at 4 weeks.

**Table 3:** Targeted qPCR analysis of mammary glands after 3, 4, or 10 weeks on HFD versus LFD

**Inflammation PCR array**		**Fold change HFD versus LFD**		
**Symbol**	**Description**	**3 wks MG**	**4 wks MG**	**10 wks MG**
*Ccl24*	*Chemokine (C-C motif) ligand 24*	2.7	-	-
*Ccl3*	*Chemokine (C-C motif) ligand 3*	2.3	-	-
*Il4*	*Interleukin 4*	2.2	-	-
*Il5ra*	*Interleukin 5 receptor, alpha*	3.0	-	-
*RANKL*	*Receptor activator of nuclear factor kappa-B ligand*	-	17	-
*Ccl1*	*Chemokine (C-C motif) ligand 1*	-	-	4.5
*Ccl17*	*Chemokine (C-C motif) ligand 17*	-	-	3.2
*Ccl19*	*Chemokine (C-C motif) ligand 19*	-	-	7.5
*Ccl20*	*Chemokine (C-C motif) ligand 20*	-	-	5.2
*Ccl22*	*Chemokine (C-C motif) ligand 22*	-	-	7.6
*Il1b*	*Interleukin 1 beta*	-	-	2.1
*Il2rb*	*Interleukin 2 receptor, beta chain*	-	-	2.7
*Cxcr2*	*Chemokine (C-X-C motif) receptor 2*	-	-	4.3
*Lta*	*Lymphotoxin A*	-	-	3.2
*Xcr1*	*Chemokine (C motif) receptor 1*	-	-	2.5

N = 3 high fat diet (HFD) mice, n = 3 low fat diet (LFD) mice; n = 3 mice with early developing tumors while on HFD (HFD-E), n = 3 LFD mice with tumors. *P* ≤0.05 for all genes listed.

At the 3- and 4-week diet time points, mice also had one inguinal mammary gland surgically cleared of endogenous mammary epithelium (cleared fat pad), whereas the contralateral gland was kept intact (intact gland). This was done to identify effects that can be mediated in the fat pad independently of epithelial cells. Analysis of mammary gland inflammatory gene expression showed that upregulated genes only overlapped with that in intact glands for *Il5ra* at 3 weeks (Table 4). At 4 weeks, however, the cleared fat pads showed robust upregulation of *Il1f6* (*Il36α*) (6.5-fold) and *Il1f8* (*Il36β*) (4.9-fold). These genes were not upregulated in intact glands.

**Table 4:** Targeted qPCR analysis of intact mammary glands and epithelium-devoid fat pads on HFD versus LFD

	**Fold change HFD versus LFD**			
**Gene**	**3 wks intact**	**3 wks cleared**	**4 wks intact**	**4 wks cleared**
*Ccl24*	2.7	-	-	-
*RANKL*	-	-	17.0	-
*Ccl3*	2.3	-	-	-
*Il4*	2.2	-	-	-
*Il5ra*	3.0	3.3	-	-
*Il1f6*	-	-	-	6.5
*Il1f8*	-	-	-	4.9

N = 3 high fat diet (HFD) mice, n = 3 low fat diet (LFD) mice. *P* ≤0.05 for all genes listed.

The Inflammatory Cytokines and Receptors PCR array identified 10 genes that were upregulated by HFD at 10 weeks on diet (Table 3). The upregulated genes were *Il1b, Il2rb, lymphotoxin α (Lta),* and a number encoding immune chemokines and chemokine receptors: *chemokine* (*C-C motif*) *ligand* (*Ccl*) 1, *Ccl17*, *Ccl19, Ccl20*, *Ccl22*, *chemokine* (*C-motif*) *receptor 1* (*Xcr1*), and *chemokine* (*C-X-C motif*) *receptor* (*Cxcr*) *2.* Similar to the Growth Factor gene analysis, there was no overlap between the genes found to be modulated at 3, 4, and 10 weeks on diet.

### Tumor characteristics

To determine the properties of HFD-E tumors that could explain their reduced latency and their enhanced proliferative characteristics, we also analyzed RNA expression by qRT-PCR using Breast Cancer, and Cancer Pathway Finder PCR arrays. This analysis (Table 5) showed upregulation of *cyclin D2* (*Ccdn2*), *insulin-like growth factor 1 receptor* (*Ifg1r*), *telomerase reverse transcriptase* (*Tert*), *Slit2*, and *β-catenin (Ctnnb1)* expression, whereas *keratin 8* (*Krt8*) and *keratin 18* (*Krt18*) were expressed at lower levels. Discordant with the pro-tumorigenic properties of many genes upregulated in HFD-E tumors, several upregulated genes are known tumor suppressors (*Brca2*[33], *Slit2*[34], *Trp53*[35], *Apaf1*[36], and *Brca1*[33]). Nonetheless, it is striking that the HFD-E tumors displayed a unique gene expression profile in comparison to LFD tumors.

**Table 5:** Targeted qPCR analysis comparing HFD-E tumors with LFD tumors

**Breast cancer genes**		**HFD-E versus LFD tumor**
**Symbol**	**Description**	**Fold change**
*Brca2*	*Breast cancer 2*	1.8
*Ccnd2*	*Cyclin D2*	3.5
*Igf1r*	*Insulin-like growth factor I receptor*	3.5
*Slit2*	*Slit homolog 2 (Drosophila)*	2.9
*Trp53*	*Transformation related protein 53*	1.6
*Krt18*	*Keratin 18*	-3.8
*Krt8*	*Keratin 8*	-4.7
**Cancer pathway finder genes**		**HFD-E versus LFD tumor**
*Apaf1*	*Apoptotic peptidase activating factor 1*	1.8
*Brca1*	*Breast cancer 1*	1.8
*Ctnnb1*	*Catenin (cadherin associated protein), beta 1*	1.8
*Tert*	*Telomerase reverse transcriptase*	1.9

N = 3 mice with early developing tumors while on high fat diet (HFD-E), n = 3 low fat diet (LFD) mice with tumors. *P* ≤0.05 for all genes listed.

### Pathway analysis

In ontology analyses (Table 6), few canonical pathways were altered at 3 weeks and 4 weeks, and among those pathways that met statistical significance criteria, few genes per pathway were significantly altered (see Additional files 7, 8). By 10 weeks, a much broader range of genes were altered and several statistically significant pathways were identified, including Granulocyte and Agranulocyte Adhesion and Diapedesis (*P* = 5.66 × 10^-9^), Hepatic Fibrosis/Hepatic Stellate Cell Activation (*P* = 1.55 × 10^-6^), Altered T Cell and B Cell Signaling in Rheumatoid Arthritis (*P* = 3.24 × 10^-6^), and Role of Macrophages, Fibroblast and Endothelial Cells in Rheumatoid Arthritis (*P* = 3.32 × 10^-6^) (see Additional file 9). The ontology analyses also identified several statistically significant pathways altered in the HFD-E tumors. These include Molecular Mechanisms of Cancer (*P* = 7.1 × 10^-7^), p53 Signaling (*P* = 7.1 × 10^-7^), Basal Cell Carcinoma Signaling (*P* = 1.27 × 10^-5^), GADD45 Signaling (*P* = 1.43 × 10^-5^), and Role of NANOG in Mammalian Embryonic Cell Pluripotency (*P* = 4.54 × 10^-5^) (see Additional file 10).

**Table 6:** Top canonical pathways of 3, 4, and 10 weeks on diet and tumor sample groups

**qPCR dataset**	**Ingenuity canonical pathways**	**B-H-adjusted *P*-value**	**Ratio**	**Molecules**
**3 weeks**	Communication between Innate and Adaptive Immune Cells	9.11E-03	2/109	CCL3L1/CCL3L3, IL4
	Granulocyte Adhesion and Diapedesis^a^	9.33E-03^a^	2/175^a^	CCL3L1/CCL3L3, CCL24
	Agranulocyte Adhesion and Diapedesis^a^	9.33E-03^a^	2/186^a^	CCL3L1/CCL3L3, CCL24
**4 weeks**	Human Embryonic Stem Cell Pluripotency	1.44E-04	3/156	BDNF, FZD5, LEFTY2
	Mouse Embryonic Stem Cell Pluripotency	4.07E-03	2/99	LIF, FZD5
	Role of NANOG in Mammalian Embryonic Stem Cell Pluripotency^a^	4.07E-03^a^	2/114^a^	LIF, FZD5
	Role of Osteoblasts, Osteoclasts and Chondrocytes in Rheumatoid Arthritis	1.20E-02	2/238	TNFSF11, FZD5
	Role of Macrophages, Fibroblasts and Endothelial Cells in Rheumatoid Arthritis^a^	1.83E-02^a^	2/332^a^	TNFSF11, FZD5
**10 weeks**	Granulocyte Adhesion and Diapedesis^a^	5.66E-09^a^	8/166^a^	IL1A, CXCR2, CCL17, CCL20, IL1B, CCL22, CCL19, CCL1
	Agranulocyte Adhesion and Diapedesis^a^	5.66E-09^a^	8/176^a^	IL1A, CXCR2, CCL17, CCL20, IL1B, CCL22, CCL19, CCL1
	Hepatic Fibrosis / Hepatic Stellate Cell Activation	1.55E-06	6/140	VEGFA, IL1A, TGFB1, TGFA, IL1B, PGF
	Altered T Cell and B Cell Signaling in Rheumatoid Arthritis	3.24E-06	5/86	IL1A, IL2, TGFB1, LTA, IL1B
	Role of Macrophages, Fibroblasts and Endothelial Cells in Rheumatoid Arthritis^a^	3.32E-06^a^	7/311^a^	VEGFA, IL1A, TGFB1, LTA, IL1B, IL7, PGF
**Tumor**	Molecular Mechanisms of Cancer	7.10E-07	7/378	TP53, CCND2, APAF1, BMP7, BRCA1, CTNNB1, BMP10
	p53 Signaling	7.10E-07	5/96	TP53, CCND2, APAF1, BRCA1, CTNNB1
	Basal Cell Carcinoma Signaling	1.27E-05	4/73	TP53, BMP7, CTNNB1, BMP10
	GADD45 Signaling	1.43E-05	3/22	TP53, CCND2, BRCA1
	Role of NANOG in Mammalian Embryonic Stem Cell Pluripotency^a^	4.54E-05^a^	4/114^a^	TP53, BMP7, CTNNB1, BMP10

^a^Pathways that are common between groups, with 10 weeks on diet, and tumor groups having an increased number of pathway molecules from 3 and 4 weeks on diet. B-H, Benjamini-Hochberg.

### Dietary effects on metabolic parameters, hormone levels and systemic factors

As shown in Additional file 3, there were no major differences in non-fasting blood insulin or blood glucose levels at 10 weeks on LFD or HFD, or in HFD-E and LFD tumor-bearing mice. Mice fed LFD and HFD had glucose levels in the normal non-fasting range for BALB/c mice (<319 mg/dL) (see Additional file 3: Figure S2A,C) [37]. Non-fasting serum insulin levels were similar at 10 weeks between LFD and HFD, with some values in the hyperinsulinemic range (normal non-fasting insulin = 0.77 ng/mL) (see Additional file 3: Figure S2B) [37]. Insulin levels in HFD-E and LFD tumor-bearing mice varied widely and generally were in the hyperinsulinemic range (see Additional file 3: Figure S2D). The high insulin values were most likely indicative of some degree of insulin resistance. There were no differences in E or P levels at 4 or 10 weeks on HFD or LFD, or in tumor bearing mice (data not shown).

Plasma levels of additional growth and inflammatory factors were measured by Ab arrays to investigate potential systemic effects of HFD exposure (Table 7). At 4 weeks on HFD, the levels of OPG and IGF-1 were significantly elevated (1.3-fold, *P* = 0.04 and 1.3-fold, *P* = 0.005, respectively); this increase was transient and not seen at later time points, or in tumor-bearing mice. At 10 weeks on HFD, the only significantly modulated systemic protein was prolactin, of which there was decreased plasma expression (0.68-fold, *P* = 0.03). However, there was a trend toward increased IL-1α levels (7.8-fold, *P* = 0.066). In HFD-E tumors, HGF was significantly increased (2.1-fold, *P* = 0.046). We compared mammary glands for mRNA expression of the factors detected in the plasma samples. The possible increase in systemic IL-1α parallels the significant increase in mammary gland *Il1a* RNA expression observed at 10 weeks on HFD (Table 3). Notably, mRNA encoding OPG, IGF-1, HGF, and prolactin were not detected at any time point in mammary gland or tumors, suggesting alternative systemic sources for their presence in plasma.

**Table 7:** Effect of diets on plasma levels of growth and inflammatory factors

	**4 wks on diet**	**10 wks on diet**	**Tumors**
	**HFD versus LFD**	**HFD versus LFD**	**HFD-E versus LFD**
**Serum factor**	**Fold change**	**Fold change**	**Fold change**
HGF	-	-	2.1
OPG	1.3	-	-
IGF-1	1.3	-	-
prolactin	-	0.68	-

N = 4 mice on high fat diet (HFD) for 4 wks, n = 4 mice on low fat diet (LFD) for 4 wks; n = 4 mice on HFD for 10 wks, n = 4 mice on LFD for 10 wks; n = 6 HFD mice with early developing tumors (HFD-E), n = 6 LFD mice with tumors. *P* ≤0.05 for all genes listed.

## Discussion

In this study we have identified the effects of HFD started during puberty, to reduce the latency of DMBA-induced mammary cancers. Most notably, tumors that developed early (HFD-E) had important characteristics that differed from those of LFD tumors that developed significantly later. We identified the likely major contributors to HFD-induced decreased latency as local and systemically increased growth factor expression, and promotion of inflammatory and angiogenic processes. This occurred without causing significant body weight gain or obesity, and the metabolic effects of HFD on blood glucose and insulin levels were modest. It is particularly noteworthy that, although DMBA mutagenesis is virtually random in its targeting of genes in tumorigenesis, the tumors arising with short latency on HFD showed a unique gene expression profile, highlighting the potent overarching influence of HFD.

### Proliferation

We previously reported that pubertal BALB/c mice fed HFD for 4 weeks showed increased mammary epithelial cell proliferation [18]. Herein, we observed that HFD induced elevated mammary gland expression of several growth factor genes as early as 3 and 4 weeks on diet. In particular, *RANKL* was robustly induced at 4 weeks. RANKL is a P-induced paracrine factor that has known mitogenic activity in the mammary gland [38-40]. Plasma levels of OPG, a decoy receptor and RANKL antagonist [41], were also elevated at this time, and this may reflect a physiologic response to excess mammary RANKL levels. Additionally, plasma levels of IGF-1 were elevated at this time. Systemic IGF-1 and mammary gland expression of *RANKL* are plausible early sources of enhanced proliferation prior to the appearance of tumors in HFD-fed BALB/c mice [28-30,42,43]. IGF-1 is an essential growth factor for TEB formation [42] that has been implicated in breast cancer progression [43].

By 10 weeks on HFD, a time point prior to the appearance of palpable tumors, mammary glands showed increased numbers of abnormal mammary epithelial lesions, and enhanced cellular proliferation. Increased levels of the RNA encoding growth factors associated with mammary epithelial cell proliferation and development were detected. Perhaps most provocative among these was the gene encoding TGFα, an EGFR ligand that has been associated with precocious alveologenesis, delayed involution, and mammary tumorigenesis [21]. Interestingly, it was recently reported that paracrine EGFR signaling between tumor-associated macrophages and murine breast cancer cells can promote a cancer stem cell-like phenotype [44]. Also provocative was the increased expression of a number of genes in the TGF-β superfamily (*Bmp2* and *3*, *Gdf5* and *10*, *Nodal*, and *Tgfb1*) in HFD-fed mice. Signaling by TGF-β1 family members mediate embryonic development, tissue homeostasis and regeneration, immune responses, tumor suppression, and metastasis, as well as govern the behavior of many stem cell populations [45-48]. TGF-β1 has an established role in mammary gland ductal development [49-51]. The Nodal signaling pathway is activated by *Cripto-1*, which encodes a growth factor with a role in mammary gland development that is capable of inducing ductal hyperplasia [52]. *Lefty2*, which was downregulated at 4 weeks, functions as an antagonist of the *Nodal* pathway [53], and this earlier downregulation may set the stage for activation of Nodal signaling at 10 weeks. Specific roles for *Bmp3* and *Gdf10* in the mammary gland have not been reported. Increased *Tert* expression is also associated with the immortalization of cells, and as such, it antagonizes apoptosis [54]. This may also contribute to a proliferative phenotype. Indeed, 95% of human cancers show increased *Tert* activity [55]. Upregulation of genes encoding IL-2 and IL-7 has been associated with some classes of breast tumors [56,57]; these were also upregulated in mammary glands of 10-week HFD-fed mice.

Analysis of HFD-E tumor characteristics showed increased cellular proliferation that was associated with upregulation of genes that promote cellular proliferation such as *Bmp7*, *Ccdn2*, *Inha*, and *Igf1r*. BMPs are recognized as key regulators during the control of cell fate and cancer development [58], and BMP signaling can downregulate levels of mitotic checkpoint components in human breast cancer cells [59]. *Bmp7* has also been implicated in increasing the metastatic potential of 4 T1 mouse mammary tumor cells [60]. There was also a trend toward upregulation of *Fgf15* (*P* = 0.09). FGFs and their receptors control a wide range of biological functions operative in cancer including cellular proliferation [61]. On the other hand, *Bmp10*, which has been shown to decrease aggressiveness of breast cancer cells [62], was downregulated in HFD-E tumors. Thus, decreased *Bmp10* expression is consistent with greater cellular proliferation of HFD-E tumors. Expression of IGF-1R is known to be increased in human breast cancers and associated with increased cellular proliferation [63-66]. Inhibins are growth factors that are also involved in cell proliferation and differentiation, and inhibin α is expressed in normal mammary tissues and in human breast cancers; however, its specific role in tumors is not well understood [67]. The increased expression of *Ccdn2* is discordant with enhanced tumorigenesis of HFD-E tumors, as loss of cyclin D2 expression is frequent in breast cancers [68]. However, transgenic overexpression of cyclin D2 does block lobuloalveolar development [69] and, perhaps, *Ccdn2* overexpression in our system could be viewed as suppressing differentiation.

Several genes that are activated in response to DNA damage (*Brca1*, *Brca2*, and *Trp53*) were upregulated in HFD-E tumors. *Trp53* is a downstream target of *Brca1*[70], and *Apaf1*, an important mediator of apoptosis that is a downstream target of *Trp53*, is also upregulated [71]. Thus, the upregulation of these genes appears to constitute the upregulation of a pathway associated with DNA damage, perhaps resulting from exposure to the mutagenic carcinogen DMBA, rather than the coincident overexpression of several mutated tumor suppressor genes. It is plausible that *Trp53* upregulation of *Apaf1* is related to removal of damaged cells by apoptosis [71]. While DMBA treatment was dissociated in time from the occurrence of tumors, DMBA is known to induce aneuploidy and unstable karyotypes that might sustain DNA damage long after exposure [72]. Additionally, expression of *Krt*8 and *18*, associated with more differentiated mammary luminal cells [73,74], was significantly decreased, suggesting a more aggressive phenotype of HFD-E tumors. In regard to increased *Ctnnb1* expression, the Wnt/β catenin pathway is involved in normal mammary gland proliferation and development, and associated with poor prognosis in breast cancer [75]. Elevated *Ctnnb1* (*β-catenin*) expression may activate this pathway.

HGF was elevated in the plasma of HFD-E tumor-bearing mice, and may play a role in driving HFD-E tumor growth. HGF regulates multiple cellular processes that stimulate cell proliferation, invasion, and angiogenesis, both in the normal mammary gland [76] and in breast cancer [77]. HGF is generally produced locally within the mammary gland/mammary cancer and acts in a paracrine manner. However, no HFD-induced increase in mammary gland or tumor expression of HGF was observed in the present study. Recently, HGF serum levels were reported to be significantly elevated with increasing tumor stage in breast cancer patients, raising the possibility of alternate sites of HGF production [78].

The integrated overview of the various aforementioned genes that is provided by pathway analysis tends to support the notion that genotoxic stress has activated p53 and Gadd45 signaling, two pathways associated with DNA damage [79,80]. The altered expression of Trp53, Ccnd2, Apaf1, Brca1, and Ctnnb1 identified the p53 signaling and GADD45 signaling pathways to high significance. Provocatively, altered expression of a partially overlapping set of genes, *Trp53*, *Bmp7*, *Ctnnb1*, and *Bmp10*, identified the Basal Cell Carcinoma signaling and Role of NANOG in Mammalian Stem Cell Pluripotency pathways. This suggests similarity between the HFD-E tumors and breast tumors displaying stem and/or progenitor cell characteristics, such as basal-like breast cancer [81]. The downregulation of luminal epithelial makers, Krt8 and 18, in HFD-E tumors is also consistent with this. Although several of the HFD-E tumors were ER-positive, basal-like breast cancers can express ERα [82]. Further, epidemiological studies have associated basal-like breast cancer with increased abdominal adiposity [83]; although HFD-E tumor-bearing animals were not obese, obesity was associated with HFD.

### Angiogenesis

It is noteworthy that a number of genes associated with angiogenesis (*Gdf5*, *Nodal*, *Pgf*, *Vegfa*, and *Cxcl12*) were upregulated by HFD at 10 weeks, concomitant with the observation of increased vascularity in the mammary glands of HFD-fed mice. *Nodal* expression is significantly elevated in malignant human breast cancers versus benign breast disease [84] and has been associated with vasculogenic mimicry, defined as functional plasticity of aggressive cancer cells forming *de novo* vascular networks [85]. GDF5 has also been shown to have angiogenic properties [86]. PGF and VEGF-A, angiogenic proteins of the VEGF family, are upregulated mainly in pathologic conditions, such as breast cancer, and are associated with poor prognosis [87]. CXCL12, produced by stromal fibroblasts within invasive breast cancers, promotes angiogenesis [88] and has additionally been associated with increased macrophage density in tumors [89]. Coincident with the induction of the above mentioned genes associated with angiogenesis, analysis of plasma at this time revealed decreased prolactin levels. Prolactin is reported to have anti-angiogenic effects [90] and its reduced expression may promote angiogenesis.

HFD-E tumors also exhibited increased blood vessel density and genes associated with angiogenesis, such as *Ntf3*, *Bmp10*, and *Slit2*, were also differentially regulated in these tumors. In fact, *Ntf3* was the most highly upregulated growth factor gene (53-fold). Neurotrophins and the neurotrophin receptor p75 are expressed in human breast cancers and are implicated in promoting angiogenesis, tumor growth, invasion, and resistance to apoptosis [91]. Interestingly, in mice fed 60% HFD, neurotrophin expression is increased in the brain, suggesting its upregulation here in HFD-E tumors may also be diet-induced [92]. Further, we found that *Bdnf*, another member of the neurotrophin family of growth factor genes [93], was robustly induced at 4 weeks on HFD, perhaps suggesting an association between this family of growth factors and HFD.

### Inflammatory processes

The induction of *Ccl24* (*eotaxin-2*) and *Ccl3* (*MIP-1α*) RNAs at 3 weeks was concomitant with influx of eosinophils into the mammary peri-epithelial compartment. Both CCL24 [94] and CCL3 [95] are potent chemoattractants for eosinophils. *Il5ra* RNA is also elevated at this time; IL-5 is a differentiation factor for eosinophil progenitors [96]. Consistent with chemotaxis of eosinophils to adipose tissue, upregulation of *Il4* RNA has also been observed [97]. *Ccl24*, *Ccl3*, and Il4 mRNAs all had high correlation with the level of eosinophils at 3 weeks on diet (*Ccl24*: *r* = 0.865 to 0.886; Ccl3: *r* = 0.667 to 0.812; Il4: *r* = 0.714 to 0.943 (depending upon mammary structural element); see Additional file 11). The induction of *Il4* RNA has potentially important implications for the function of macrophages in the mammary gland, as eosinophil-derived IL-4 is essential for the maintenance of alternatively activated (that is, M2) macrophages in adipose tissue [97]. This is consistent with the high numbers of Arg1^+^ macrophages observed in the peri-epithelial stroma of the normal gland at 3 and 4 weeks on diet, and in the vicinity of early lesions and within tumors in DMBA/HFD-treated mice. While pathway analysis did not identify any canonical pathways to a high level of significance, chemotaxis of eosinophils was identified as a highly significant function (*P* = 6.4 × 10^-5^) (see Additional file 7), and eosinophil levels correlated well with RNAs for all of the aforementioned chemokines and cytokine (see Additional file 11). Another inflammatory growth factor RNA increased in mice at 4 weeks was that encoding RANKL, a potent activator of NF-κB [98]. Thus, RANKL may play a role in the upregulation of pro-inflammatory factors at this time. In parallel to the analysis of whole intact mammary gland at 3 and 4 weeks of diet, a similar analysis of epithelium-devoid fat pads contralateral to the intact mammary glands was carried out. At 3 weeks, this revealed that *Il5r*a was similarly induced by HFD in both cleared fat pads and intact glands, suggesting that it is, in fact, associated with the stroma. At 4 weeks, this analysis also revealed HFD induction of the genes encoding IL-1f6 (IL-36α) and IL-1f8 (IL-36β), IL-1 family cytokines known to be expressed in adipocytes as inflammatory mediators [99]. This induction is not observed in intact glands. It may be that the mammary epithelium suppresses this induction in intact glands, or, alternatively, that the stromal induction of these cytokines is obscured in the intact gland by dilution or because the same cytokines are downregulated in the epithelium, while being upregulated in the stroma. This highlights the mammary stroma as an epithelium-independent source of inflammatory factors that can mediate the effects of HFD.

At 10 weeks on diet, the RNA encoding a number of cytokines and chemokines associated with immune function were modulated by HFD, and these may have profound implications for the status of the immune system at this point in time. Most interesting are the genes encoding the chemokines CCL1, CCL17, and CCL22, as well as the cytokine TGF-β1, which are all associated with the recruitment and function of immunosuppressive Treg cells [100-102]. There is also one report of CCL20 recruiting Treg cells in an induced colorectal cancer model [103]. Further, these factors are all products of M2 macrophages, recruitment of which was induced by HFD at 10 weeks of diet. In fact, TGF-β1 additionally can function in the generation of immunosuppressive M2 macrophages [104], perhaps, in part, through the suppression of M1 macrophage activity [105]. Upregulation of *Ccl19*, *Ccl20* and *chemokine* (*C-X-C motif*) *receptor* (*Cxcr*) *2* has also been associated with breast tumor growth and invasion [106-108], *Cxcr2* particularly with angiogenic processes [108]. Systemic levels of prolactin were also reduced at 10 weeks on HFD. As prolactin can suppress the function of suppressor T cells [109], a decrease in its expression would be expected to enhance Treg activity. Indeed, increased numbers of Treg cells have been associated with increased DMBA-induced mammary carcinogenesis in mice [110], suggesting the importance of Treg-associated chemokines in this experimental system. Characterization of the influence of dietary fat upon T cell populations awaits future studies.

**Figure 4:**
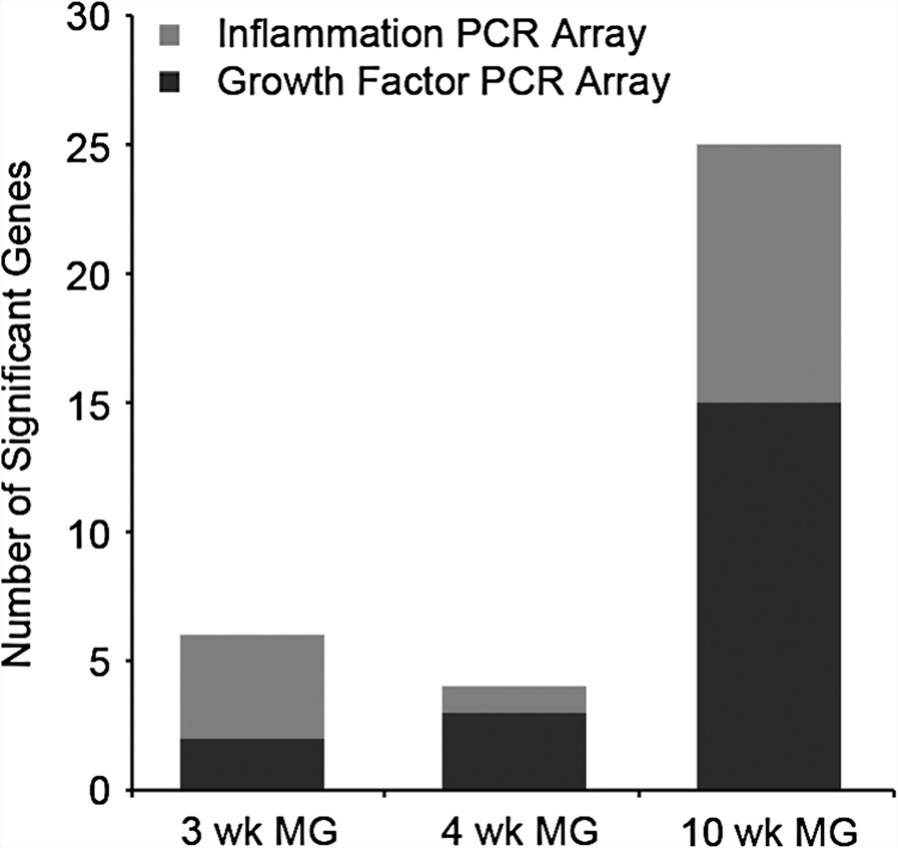
**Increase in significant genes identified by targeted pathway PCR arrays between 4 and 10 weeks on diet.** RNA isolated from week 3, 4, and 10 diet groups were analyzed using Growth Factors and Inflammatory Cytokines and Receptors PCR arrays (SABiosciences). The week-3 sample group identified six significant genes, the week-4 sample group identified five significant genes, and the week-10 diet group identified twenty-five significant genes.

Pathway analysis comparing mice on HFD to those on LFD at week 10 identified Granulocyte and Agranulocyte Adhesion and Diapedesis (identified by *Il1a, Cxcr2, Ccl17, Ccl20, Il1b, Ccl22, Ccl19,* and *Ccl1*), which is consistent with leukocyte migration into the mammary gland. Furthermore, immune cell trafficking (adhesion of immune cells and leukocyte migration) was among functions showing the highest significance at 10 weeks on HFD (*P* = 1.64 × 10^-13^ and 2.03 × 10^-13^, respectively). Pathway analysis also identified Altered T Cell and B Cell Signaling in Rheumatoid Arthritis (identified by altered expression of *Il1a*, *Il2*, *Tgfb1*, *Lta*, and *Il1b*) and Role of Macrophages, Fibroblasts and Endothelial cells in Rheumatoid Arthritis (identified by altered expression of *Vegfa*, *Il1a*, *Tgfb1*, *Lta*, *Il1b*, *Il7*, and *Pgf*) (see Additional file 9), pathways that can lead to macrophage-mediated pathology [111] and abnormal angiogenesis [112]. The identification of the Hepatic Fibrosis/Hepatic Stellate Cell Activation pathway (identified by *Vegfa*, *Il1a*, *Tgfb1*, *Tgfa*, *Il1b*, and *Pgf*) is interesting, as the polarization of M2 macrophages is a critical component of this pathology [113]. The levels of RNA encoding IL-1b, a common factor in all of these pathways, correlated well with the level of macrophages in the mammary gland (*r* = 0.828 to 0.886 (depending upon mammary structural element); see Additional file 11) suggesting the plausibility that these pathways may operate, at least in part, through macrophage recruitment. The genes included on the PCR arrays were highly selected for genes with established involvement in particular pathways (that is, growth factors, inflammation). Thus, it is also noteworthy to observe that the absolute number of significant genes on each targeted pathway array increased between week 4 and week 10 (Figure 4). The parallel increase in expression of inflammation and growth factor genes underscores that immune infiltrates can alter the expression of cytokines with regulatory effects on mammary epithelial proliferation.

The status of inflammatory processes also differed in HFD-E tumors compared to LFD tumors. There was a significant increase in intra-tumoral and stromal alternatively activated M2 (Arg1+) macrophages. The M2 phenotype is associated with tumor-associated macrophages that are known to promote the growth of tumors through support of angiogenic and tissue remodeling processes, as well as immune suppression [114]. The increase in M2 macrophages is consistent with the enhanced vascularity of HFD-E tumors in comparison to LFD tumors. While none of the cytokines identified as expressed in HFD-E tumors is known to generate M2 macrophages, *Il4* is upregulated at 3 weeks and *Tgfb1* is upregulated at 10 weeks on HFD, and these cytokines may be involved in the prior polarization of M2 macrophages [104]. There is compelling evidence at the molecular level that many cancers, including breast cancer, are linked to a dysregulated inflammatory response [115]. The present results that demonstrate HFD-induced inflammatory processes involving M2 macrophages are consistent with a role for these macrophages and their secreted chemokines/cytokines in the promotion of HFD-E tumors.

As for the role of HFD, there is evidence that it can induce low-grade inflammation after feeding [116]. It has been proposed that HFD induces post-prandial gut permeability allowing low levels of bacterial endotoxin from the gut to enter the circulation and, thus, induce low-grade inflammation [116,117]. There is also evidence that saturated fatty acids can directly modulate inflammatory processes through toll-like receptor 4 (TLR4) [118]. Palmitic acid, an abundant component of lard [119], has particularly been implicated in TLR2 [120,121] and TLR4 signaling [120-123]. This stimulation has been associated with increased IL-1 signaling [120]. This is consistent with our observation of increased levels of *Il1a* and *Il1b* gene expression after 10 weeks on HFD.

### Systemic metabolic effects of HFD

The levels of metabolism-related factors such glucose, insulin, leptin, and resistin were not significantly altered by HFD. However, an inflammatory growth factor RNA that increased in HFD mice at 4 weeks was that encoding LIF, an IL-6-class cytokine that can stimulate lipolysis and fat loss [124-126]. This places LIF as a plausible mediator contributing to the lack of weight gain in BALB/c mice placed on HFD. At 10 weeks on HFD, plasma IL-1α levels were increased. Since HFD also increased mammary gland IL-1α mRNA expression at 10 weeks, increased plasma levels might reflect spillover from the mammary gland or other tissues affected by HFD. Palmitic acid has been associated with increased IL-1 signaling [120], and elevation of serum IL-1α has been reported in HFD-induced obesity in mice [127]. Tumors showed a trend toward increased *Fgf15* expression. FGF15-specific signaling is thought to control metabolic homeostasis associated with HFD to restore glucose tolerance and insulin sensitivity [128].

Other studies of the effect of dietary fat on normal mammary gland development and tumorigenesis have been carried out in rodents [129-131]. However, they differ significantly from the current studies with regard to species studied (rat versus mouse), differences in fat formulations (corn oil versus lard), caloric content (isocaloric versus non-isocaloric), obesogenic versus non-obesogenic outcomes, development of metabolic syndrome, and age at diet initiation. More similar studies on the effect of HFD on tumor development without the confounding effects of obesity have been performed in other mouse mammary cancer models. Results vary by tumor model and age at diet initiation. In two studies of the effects of HFD initiated at 4 weeks of age in mice overexpressing HER2/Neu in the mammary gland [14,15], HFD promoted tumor development by increasing tumor incidence, but without increasing tumor cell proliferation; there was only a small increase in body weight and no insulin resistance or hyperinsulinemia. In another study, HER2/Neu transgenic mice fed HFD starting in adulthood, at 10 weeks of age, showed no difference in tumor latency, incidence, or metastasis [132]. In a tumor transplant model, mice were started on HFD at 4 weeks of age, and after 16 weeks on diet, 4 T1 mammary carcinoma cells were transplanted into their mammary glands [133]. There were only slight increases in body weight with HFD. However, tumor weight and number of metastases were significantly increased by HFD. Thus, based on the results of the present study and of the previous studies of HFD initiated in pubertal mice (4 weeks of age), there was a significant promotional effect on tumor development with only a modest effect on weight gain or metabolic parameters. This is in contrast to the lack of a promotional effect of HFD when initiated in adult, 10-week-old mice. The most extensive study was carried out in the 4 T1 tumor transplant model with diet started at 4 weeks of age [133]. Similar to our present results, that study revealed an association of HFD with increased macrophage infiltration, angiogenesis, and cellular proliferation, as well as increased levels of a number of inflammatory factors.

## Conclusions

Taken together, our results demonstrate that exposure to HFD in the peri-pubertal period, and the sensitivity of the pubertal gland to HFD, initiate a sequence of inflammatory, angiogenic, and growth-promoting effects starting as early as 3 weeks on diet, which can lead to the promotion of mammary cancer development in adulthood. Notably, the observed effects of HFD were independent of significant weight gain. Importantly, this indicates a potential risk from HFD for a broader segment of the population than only those who become obese. The observation of a distinct expression profile associated with the HFD-dependent shorter latency of DMBA tumors highlights the potent influence of HFD in the context of a virtually randomly targeted carcinogen. It is also particularly noteworthy that, in our DMBA model and other tumor models, there is significant promotion of tumorigenesis under the potent influence of HFD. Future studies are needed to identify the specific effects of exposure to HFD at puberty versus adulthood, as well as to identify the mechanistic basis for dietary fat effects and potential interventions to prevent the promotional consequences of HFD exposure.

## Abbreviations

Ab: Antibody; ANOVA: Analysis of variance; Areg: Amphiregulin; Arg1: Arginase 1; B-H: Benjamini-Hochberg; BMI: Body mass index; BMP: Bone morphogenetic protein; BrdU: 5-bromo-2’-deoxyuridine; BSA: Bovine serum albumin; Ccl: Chemokine (C-C motif) ligand; Cxcl: chemokine (C-X-C motif) ligand; DAB: 3,3-diaminobenzidine; DAPI: 4’,6-diamidino-2-phenylindole; DMBA: 7,12-dimethylbenz(a)anthracene; E: Estrogen; EGFR: Epidermal growth factor receptor; ELISA: Enzyme-linked immunosorbent assay; ER: Estrogen receptor; FGF: Fibroblast growth factor; GDF: Growth differentiation factor; H2O: Water; H2O2: Hydrogen peroxide; H&E: Hematoxylin and eosin; HFD: High fat diet; HFD-E: Early developing tumors on high fat diet; HGF: Hepatocyte growth factor; IgG: Immunoglobulin G; IL: Interleukin; LFD: Low fat diet; LIF: Leukemia inhibitory factor; MG: Mammary gland; OPG: Osteoprotegerin; P: Progesterone; PBS: Phosphate-buffered saline; PBSA 1% bovine serum albumin in phosphate-buffered saline; PCR: Polymerase chain reaction; psi: Pounds per square inch; qRT-PCR: Quantitative reverse transcription-polymerase chain reaction; RANKL: Receptor activator of nuclear factor kappa-B ligand; RT: Room temperature; SEM: Standard error of the mean; TEB: Terminal end bud; TGF: Transforming growth factor; TLR: Toll-like receptor; TNF: Tumor necrosis factor; Treg: Regulatory T cell; VEGF: Vascular endothelial growth factor.

## Competing interests

The authors declare that they have no competing interests.

## Authors’ contributions

YZ and YST carried out the majority of the experiments. MDA carried out experiments and assisted in writing the manuscript. IML performed the histopathological analysis of the tumors. ELK and MAT contributed to pathway analysis and interpretation of gene expression analyses. RCS and SZH conceived and designed the study, interpreted the results and wrote the manuscript. All authors read and approved the final manuscript.

## Supplementary Material

Additional file 1: Table S1 Diet formulations. Click here for file

Additional file 2: Figure S1 Comparison of weight gains on Diets I and II. BALB/c mice were started on high fat diet (HFD) and low fat diet (LFD) I or II at 3 weeks of age and continued until 45 weeks of age. There were virtually identical weight gains on both diets. The dips in weight between 6 and 9 weeks were due to the response to 7,12-dimethylbenz(a)anthracene (DMBA) treatments. Click here for file

Additional file 3: Figure S2 Comparison of the effects of Diets I and II on non-fasting blood levels of glucose and insulin. BALB/c mice were started on high fat diet (HFD) or low fat diet (LFD) I or II at 3 weeks of age. Blood levels of **(A,C)** glucose and **(B,D)** insulin were measured at 10 weeks on diet **(A,B)** or in tumor-bearing mice **(C,D)**. The bars represent the mean ± standard error of the mean for samples from five animals per diet at 10 weeks on diet, six early developing tumors on HFD (HFD-E), and five LFD tumor-bearing mice. ^*^P = 0.02 HFDII blood glucose level higher than LFD II at 10 weeks on diet. Click here for file

Additional file 4: Figure S3 Comparison of the effects of diets I (A) and II (B) on time-course of tumor development. Tumor development was monitored starting after the last 7,12-dimethylbenz(a)anthracene (DMBA) treatment. Kaplan-Meier plots show no significant differences in latency of tumors developing on **(A)** low fat diet (LFD) I versus LFD II or **(B)** high fat diet (HFD) I versus HFD II. Development of tumors in LFD-fed mice was observed only after 18 weeks post-DMBA treatment on either diet I or II. Time = days post last DMBA treatment. Click here for file

Additional file 5: Table S2 Tumor incidence. Click here for file

Additional file 6: Figure S4 Diagram of experimental design. Click here for file

Additional file 7: Table S3 Three weeks on diet qPCR Ingenuity Pathway Analysis. Click here for file

Additional file 8: Table S4 Four weeks on diet qPCR Ingenuity Pathway Analysis. Click here for file

Additional file 9: Table S5 Ten weeks on diet qPCR Ingenuity Pathway Analysis. Click here for file

Additional file 10: Table S6 Tumor qPCR Ingenuity Pathway Analysis. Click here for file

Additional file 11: Figure S5 Correlations between cytokine mRNA expression and leukocyte recruitment. Cycle threshold (Ct) values for the indicated mRNA in individual animals were normalized to the average Ct values for *GusB*, *Hprt1*, *Hsp90ab1*, *Gapdh*, and *Actb* mRNA in the same animals, and plotted against the indicated leukocyte counts in the same animals in association with the indicated individual structures. Correlations (*r*) were calculated by Spearman’s method. Click here for file
