# Supplementary material for: Pubertal high fat diet: effects on mammary cancer development
Source: Breast Cancer Res. 2013 Oct 25;15(5):R100. doi: 10.1186/bcr3561 (PMC3978633; doi:10.1186/bcr3561)
Supplement: Additional file 1: Table S1 — Diet formulations. [file bcr3561-S1.pdf]

Supplemental Table 1. Diet Formulations

Diet I

Ingredients (g/100 g)		Low Fat Diet	High Fat Diet
Fat	Corn Oil	4.731	6.3715
	Lard	0.4257	28.315
Carbohydrate	Dextrin	48.6172	18.9872
	Sucrose	16.558	6.3715
Protein	Casein	19.87	26.76
	DL Methionine	0.1419	0.1911
Fiber	Cellulose	1.4193	1.9114
	Inulin	1.4193	1.9114
Vitamins	RP Vitamin Mix	1.8924	2.5486
	Choline Cloride	0.1892	0.2548
Minerals	RP Mineral Mix #10	4.731	6.371
**Energy**			
kcal density/g		3.87	5.21
% kcal	Fat	12	59.9
	Carbohydrate	69.3	21.4
	Protein	18.6	18.6

Low fat diets contained a yellow die, whereas high fat diets contained a blue dye. Diet I: TestDiet, PMI Nutrition International LLC, St. Louis, MO

Diet II

Ingredients (g/100 g)		Low Fat Diet	High Fat Diet
Fat	Corn Oil	2.369	16.1498
	Lard	1.8957	31.6537
Carbohydrate	Corn Starch	54.407	8.888
	Maltodextrin	11.848	16.1498
Protein	Casein	18.987	25.8397
	L-cystine	0.2843	0.3876
Fiber	Cellulose	4.7393	6.4599
Vitamins	Vitamin Mix V10001	0.9479	1.2919
	Choline Bitartrate	0.1896	0.2584
Minerals	Mineral Mix S10026	0.9479	0.1286
	DiCalcium Phosphate	1.2322	1.6795
	Calcium Carbonate	0.5213	0.7106
	Potassium Citrate, 1 H20	1.5639	2.1318
**Energy**			
kcal density/g		3.8	5.2
% kcal	Fat	10	60
	Carbohydrate	70	20
	Protein	20	20

Diet II: Research Diets, Inc. New Brunswick, NJ
