# Supplementary material for: Pubertal high fat diet: effects on mammary cancer development
Source: Breast Cancer Res. 2013 Oct 25;15(5):R100. doi: 10.1186/bcr3561 (PMC3978633; doi:10.1186/bcr3561)
Supplement: Additional file 2: Figure S1 — Comparison of weight gains on Diets I and II. BALB/c mice were started on high fat diet (HFD) and low fat diet (LFD) I or II at 3 weeks of age and continued until 45 weeks of age. There were virtually identical weight gains on both diets. The dips in weight between 6 and 9 weeks were due to the response to 7,12-dimethylbenz(a)anthracene (DMBA) treatments. [file bcr3561-S2.pdf]

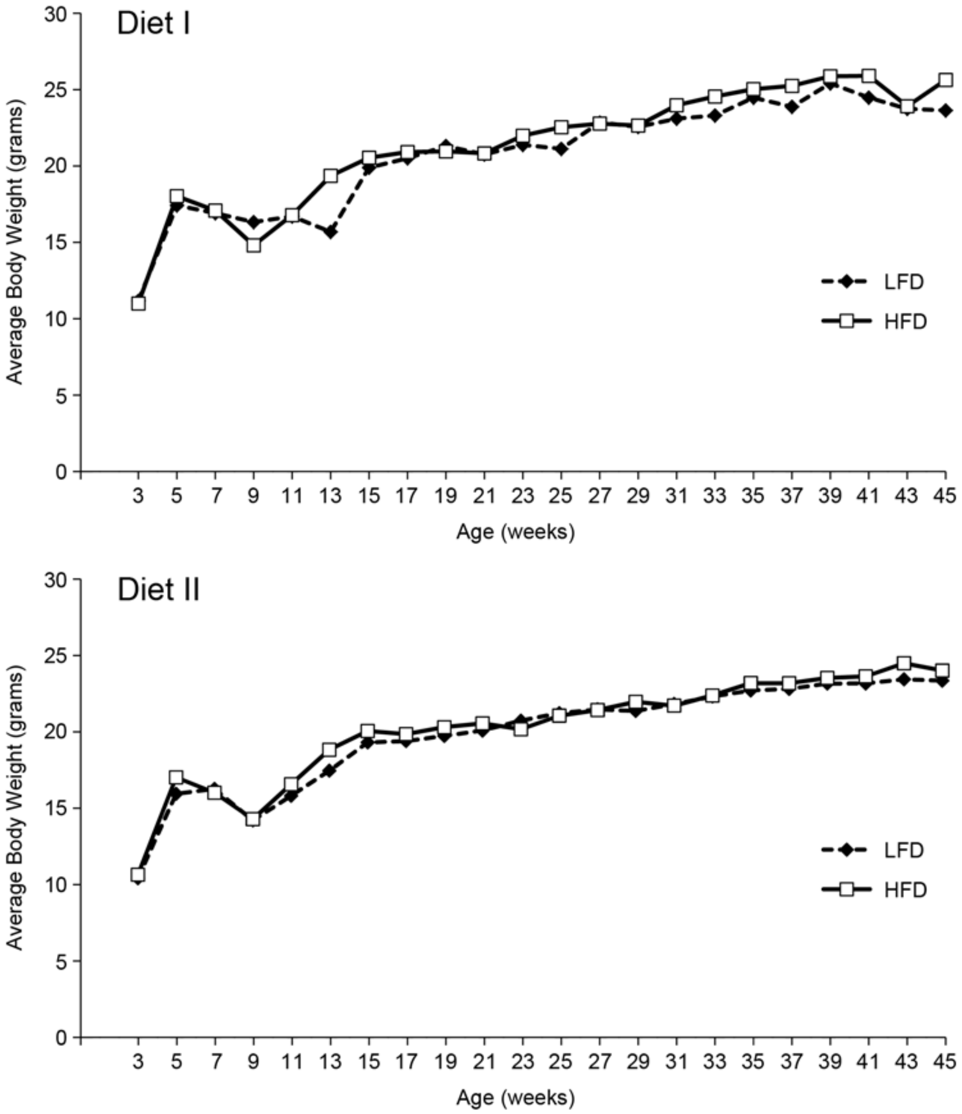

Figure S1. Comparison of weight gains on Diets I and II. BALB/c mice were started on high fat diet (HFD) and low fat diet (LFD) I or II at 3 weeks of age and continued until 45 weeks of age. There were virtually identical weight gains on both diets. The dips in weight between 6 and 9 weeks were due to the response to 7,12-dimethylbenz[a]anthracene (DMBA) treatments.
