# Supplementary material for: Pubertal high fat diet: effects on mammary cancer development
Source: Breast Cancer Res. 2013 Oct 25;15(5):R100. doi: 10.1186/bcr3561 (PMC3978633; doi:10.1186/bcr3561)
Supplement: Additional file 3: Figure S2 — Comparison of the effects of Diets I and II on non-fasting blood levels of glucose and insulin. BALB/c mice were started on high fat diet (HFD) or low fat diet (LFD) I or II at 3 weeks of age. Blood levels of (A,C) glucose and (B,D) insulin were measured at 10 weeks on diet (A,B) or in tumor-bearing mice (C,D). The bars represent the mean ± standard error of the mean for samples from five animals per diet at 10 weeks on diet, six early developing tumors on HFD (HFD-E), and five LFD tumor-bearing mice. *P = 0.02 HFDII blood glucose level higher than LFD II at 10 weeks on diet. [file bcr3561-S3.pdf]

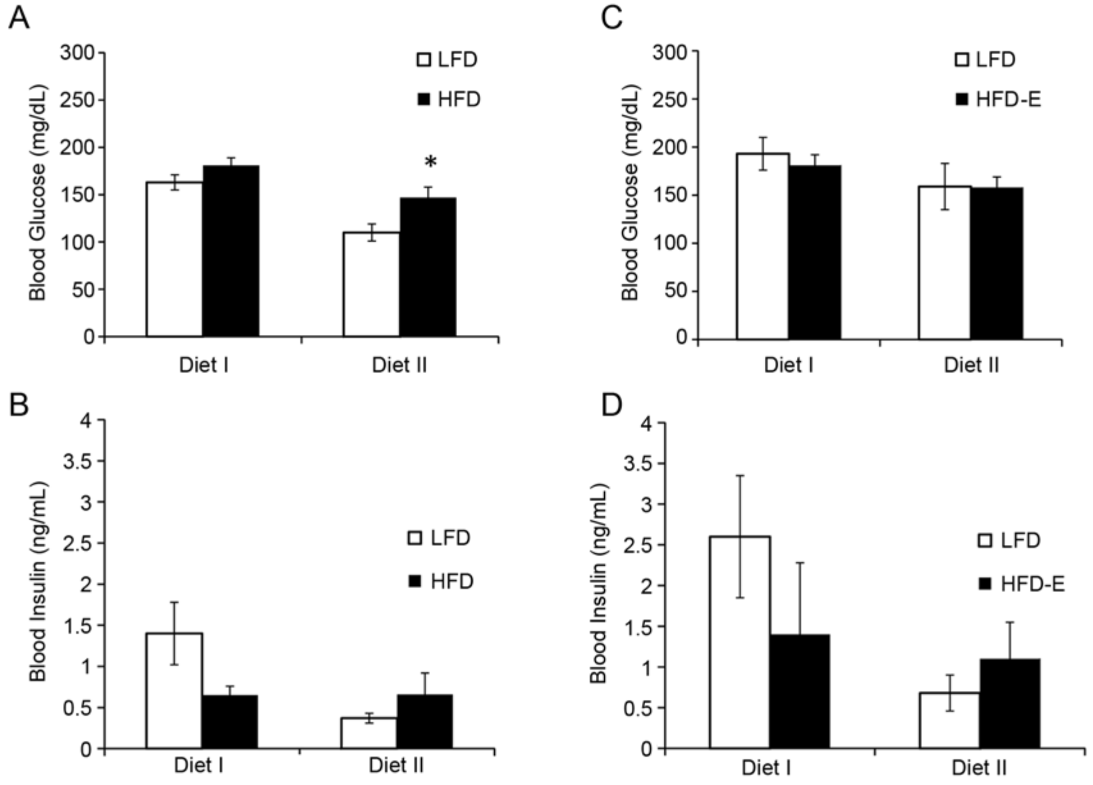

Figure S2. Comparison of the effects of diets I and II on non-fasting blood levels of glucose and insulin. BALB/c mice were started on high fat diet (HFD) or low fat diet (LFD) I or II at 3 weeks of age. Blood levels of (A,C) glucose and (B,D) insulin were measured at 10 weeks on diet (A,B) or in tumor-bearing mice (C,D). The bars represent the mean ± S.E.M. for samples from 5 animals per diet at 10 weeks on diet, 6 early developing tumors on HFD (HFD-E), and 5 LFD tumor-bearing mice.
*, p=0.02 HFD II blood glucose level higher than LFD II at 10 weeks on diet.
