# Supplementary material for: Pubertal high fat diet: effects on mammary cancer development
Source: Breast Cancer Res. 2013 Oct 25;15(5):R100. doi: 10.1186/bcr3561 (PMC3978633; doi:10.1186/bcr3561)
Supplement: Additional file 4: Figure S3 — Comparison of the effects of diets I (A) and II (B) on time-course of tumor development. Tumor development was monitored starting after the last 7,12-dimethylbenz(a)anthracene (DMBA) treatment. Kaplan-Meier plots show no significant differences in latency of tumors developing on (A) low fat diet (LFD) I versus LFD II or (B) high fat diet (HFD) I versus HFD II. Development of tumors in LFD-fed mice was observed only after 18 weeks post-DMBA treatment on either diet I or II. Time = days post last DMBA treatment. [file bcr3561-S4.pdf]

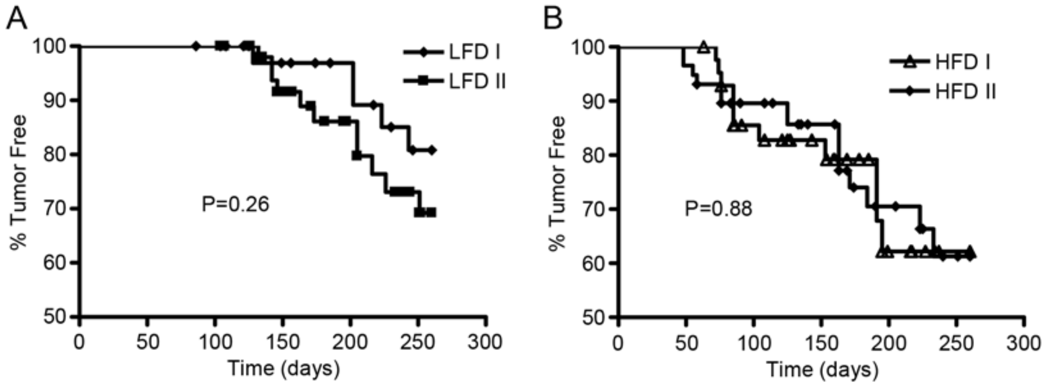

Figure S3. Comparison of the effects of diets I (A) and II (B) on tumor development. Tumor development was monitored starting after the last 7,12-dimethylbenz[a]anthracene (DMBA) treatment. Kaplan-Meier plots show no significant differences in latency of tumors developing on (A) low fat diet (LFD) I vs. LFD II or (B) high fat diet (HFD) I vs. HFD II . Development of tumors in LFD-fed mice was observed only after 18 weeks post-DMBA treatment on either diet I or II. Time = days post last DMBA treatment.
