# Supplementary material for: Pubertal high fat diet: effects on mammary cancer development
Source: Breast Cancer Res. 2013 Oct 25;15(5):R100. doi: 10.1186/bcr3561 (PMC3978633; doi:10.1186/bcr3561)
Supplement: Additional file 5: Table S2 — Tumor incidence. [file bcr3561-S5.pdf]

Supplemental Table 2. Tumor Incidence

Treatment	Tumor Incidence (N)[a]
LFD I	13.5% (37)
HFD I	26.5% (41)
LFD II	16% (50)
HFD II	25.9% (54)

Mice were treated with diet starting at 3 weeks of age, treated with DMBA, and followed for tumor development until 45 weeks of age.
[a] N = number of mice per group
