# Supplementary figures and images for: Pubertal high fat diet: effects on mammary cancer development
Source: Breast Cancer Res. 2013 Oct 25;15(5):R100. doi: 10.1186/bcr3561 (PMC3978633; doi:10.1186/bcr3561)

**Experimental Design**

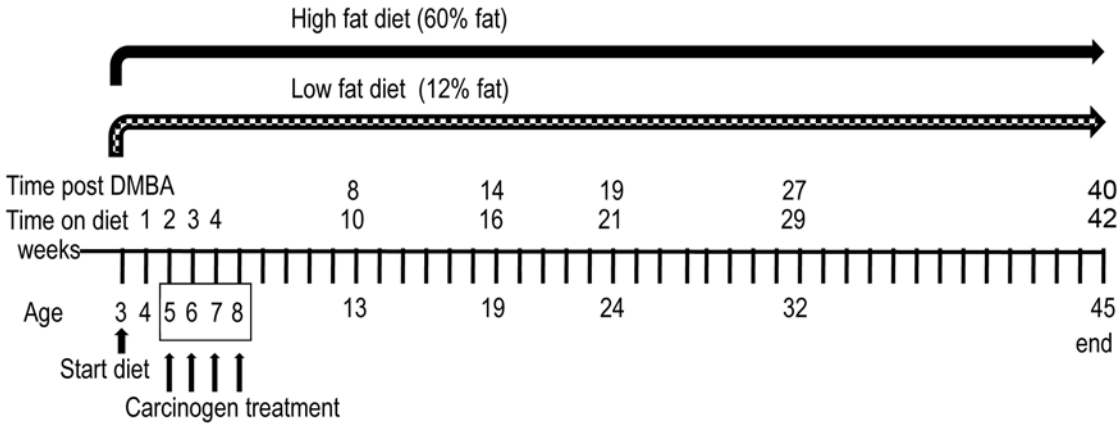

**Figure S4. Diagram of experimental design.**

Supplement: Additional file 6: Figure S4 — Diagram of experimental design. [file bcr3561-S6.pdf]
