# Supplementary material for: Pubertal high fat diet: effects on mammary cancer development
Source: Breast Cancer Res. 2013 Oct 25;15(5):R100. doi: 10.1186/bcr3561 (PMC3978633; doi:10.1186/bcr3561)
Supplement: Additional file 7: Table S3 — Three weeks on diet qPCR Ingenuity Pathway Analysis. [file bcr3561-S7.pdf]

Supplemental Table 3. 3 weeks on diet qPCR Ingenuity Pathway Analysis

1) Significant Gene List (5):

CCL24
CCL3
FGF18
Hand2
IL4

2) Top Canonical Pathways

Ingenuity Canonical Pathways	B-H Adjusted p-value	Ratio	Molecules
**Communication between Innate and Adaptive Immune Cells**	**9.11E-03**	**2/109**	**CCL3L1/CCL3L3,IL4**
**Granulocyte Adhesion and Diapedesis**	**9.33E-03**	**2/175**	**CCL3L1/CCL3L3,CCL24**
**Agranulocyte Adhesion and Diapedesis**	**9.33E-03**	**2/186**	**CCL3L1/CCL3L3,CCL24**

3) Function Table

Category	Functions Annotation	B-H Adjusted p-value	Molecules	Number of Molecules
3wk qPCR IPA Function Table				
Behavior	behavior	6.64E-03	CCL3L1/CCL3L3,HAND2,IL4	3
Cancer	hyperplasia	5.24E-03	CCL24,CCL3L1/CCL3L3,IL4	3
Cardiovascular System Development and Function	angiogenesis	1.24E-03	CCL24,FGF18,HAND2,IL4	4
	development of cardiovascular system	7.34E-03	CCL24,HAND2,IL4	3
Cell Death and Survival	necrosis	9.00E-03	CCL3L1/CCL3L3,FGF18,HAND2,IL4	4
	cell viability	1.00E-02	FGF18,HAND2,IL4	3
Cell Signaling*	flux of Ca2+	1.75E-03	CCL24,CCL3L1/CCL3L3,IL4	3
	quantity of Ca2+	3.13E-03	CCL24,CCL3L1/CCL3L3,IL4	3
Cell-mediated Immune Response	cell movement of T lymphocytes	1.24E-03	CCL24,CCL3L1/CCL3L3,IL4	3
Cell-To-Cell Signaling and Interaction*	recruitment of eosinophils	7.92E-05	CCL24,CCL3L1/CCL3L3,IL4	3
	communication of cells	2.06E-03	CCL24,FGF18,IL4	3
Cellular Development*	differentiation of connective tissue cells	6.77E-04	CCL3L1/CCL3L3,FGF18,HAND2,IL4	4
	proliferation of hematopoietic cells	1.61E-03	CCL24,CCL3L1/CCL3L3,IL4	3
Cellular Function and Maintenance	flux of Ca2+	1.75E-03	CCL24,CCL3L1/CCL3L3,IL4	3
Cellular Growth and Proliferation*	formation of osteoclasts	2.44E-04	CCL3L1/CCL3L3,FGF18,IL4	3
	proliferation of hematopoietic cells	1.61E-03	CCL24,CCL3L1/CCL3L3,IL4	3

	colony formation of cells	3.30E-03	CCL24,CCL3L1/CCL3L3,IL4	3
	proliferation of connective tissue cells	3.99E-03	CCL24,FGF18,IL4	3
	**proliferation of cells**	**4.84E-03**	**CCL24,CCL3L1/CCL3L3,FGF18,HAND2,IL4**	**5**
Cellular Movement*	**chemotaxis of eosinophils**	**6.40E-05**	**CCL24,CCL3L1/CCL3L3,IL4**	**3**
	recruitment of eosinophils	7.92E-05	CCL24,CCL3L1/CCL3L3,IL4	3
	migration of granulocytes	6.77E-04	CCL24,CCL3L1/CCL3L3,IL4	3
	cell movement of dendritic cells	6.77E-04	CCL24,CCL3L1/CCL3L3,IL4	3
	chemotaxis of mononuclear leukocytes	9.71E-04	CCL24,CCL3L1/CCL3L3,IL4	3
	cell movement of T lymphocytes	1.24E-03	CCL24,CCL3L1/CCL3L3,IL4	3
	cell movement of neutrophils	1.75E-03	CCL24,CCL3L1/CCL3L3,IL4	3
	chemotaxis of phagocytes	1.87E-03	CCL24,CCL3L1/CCL3L3,IL4	3
Connective Tissue Development and Function	formation of osteoclasts	2.44E-04	CCL3L1/CCL3L3,FGF18,IL4	3
Connective Tissue Disorders	rheumatoid arthritis	4.97E-03	CCL24,CCL3L1/CCL3L3,IL4	3
Gene Expression	activation of DNA endogenous promoter	7.54E-03	CCL3L1/CCL3L3,HAND2,IL4	3
Hematological Disease	eosinophilic inflammation	1.17E-04	CCL24,CCL3L1/CCL3L3,IL4	3
Hematological System Development and Function	**chemotaxis of eosinophils**	**6.40E-05**	**CCL24,CCL3L1/CCL3L3,IL4**	**3**
	recruitment of eosinophils	7.92E-05	CCL24,CCL3L1/CCL3L3,IL4	3
	migration of granulocytes	6.77E-04	CCL24,CCL3L1/CCL3L3,IL4	3
	cell movement of dendritic cells	6.77E-04	CCL24,CCL3L1/CCL3L3,IL4	3
	chemotaxis of mononuclear leukocytes	9.71E-04	CCL24,CCL3L1/CCL3L3,IL4	3
	cell movement of T lymphocytes	1.24E-03	CCL24,CCL3L1/CCL3L3,IL4	3
	accumulation of leukocytes	1.61E-03	CCL24,CCL3L1/CCL3L3,IL4	3
	proliferation of hematopoietic cells	1.61E-03	CCL24,CCL3L1/CCL3L3,IL4	3
	cell movement of neutrophils	1.75E-03	CCL24,CCL3L1/CCL3L3,IL4	3
	chemotaxis of phagocytes	1.87E-03	CCL24,CCL3L1/CCL3L3,IL4	3
	quantity of granulocytes	1.98E-03	CCL24,CCL3L1/CCL3L3,IL4	3

Hematopoiesis	proliferation of hematopoietic cells	1.61E-03	CCL24,CCL3L1/CCL3L3,IL4	3
Hypersensitivity Response	**chemotaxis of eosinophils**	**6.40E-05**	**CCL24,CCL3L1/CCL3L3,IL4**	**3**
	recruitment of eosinophils	7.92E-05	CCL24,CCL3L1/CCL3L3,IL4	3
Immune Cell Trafficking	**chemotaxis of eosinophils**	**6.40E-05**	**CCL24,CCL3L1/CCL3L3,IL4**	**3**
	recruitment of eosinophils	7.92E-05	CCL24,CCL3L1/CCL3L3,IL4	3
	migration of granulocytes	6.77E-04	CCL24,CCL3L1/CCL3L3,IL4	3
	cell movement of dendritic cells	6.77E-04	CCL24,CCL3L1/CCL3L3,IL4	3
	chemotaxis of mononuclear leukocytes	9.71E-04	CCL24,CCL3L1/CCL3L3,IL4	3
	cell movement of T lymphocytes	1.24E-03	CCL24,CCL3L1/CCL3L3,IL4	3
	accumulation of leukocytes	1.61E-03	CCL24,CCL3L1/CCL3L3,IL4	3
	cell movement of neutrophils	1.75E-03	CCL24,CCL3L1/CCL3L3,IL4	3
	chemotaxis of phagocytes	1.87E-03	CCL24,CCL3L1/CCL3L3,IL4	3
Immunological Disease	eosinophilic inflammation	1.17E-04	CCL24,CCL3L1/CCL3L3,IL4	3
	rheumatoid arthritis	4.97E-03	CCL24,CCL3L1/CCL3L3,IL4	3
Inflammatory Disease	eosinophilic inflammation	1.17E-04	CCL24,CCL3L1/CCL3L3,IL4	3
	rheumatoid arthritis	4.97E-03	CCL24,CCL3L1/CCL3L3,IL4	3
Inflammatory Response	**chemotaxis of eosinophils**	**6.40E-05**	**CCL24,CCL3L1/CCL3L3,IL4**	**3**
	cell movement of dendritic cells	6.77E-04	CCL24,CCL3L1/CCL3L3,IL4	3
	chemotaxis of mononuclear leukocytes	9.71E-04	CCL24,CCL3L1/CCL3L3,IL4	3
	accumulation of leukocytes	1.61E-03	CCL24,CCL3L1/CCL3L3,IL4	3
	cell movement of neutrophils	1.75E-03	CCL24,CCL3L1/CCL3L3,IL4	3
	chemotaxis of phagocytes	1.87E-03	CCL24,CCL3L1/CCL3L3,IL4	3
Molecular Transport	flux of Ca2+	1.75E-03	CCL24,CCL3L1/CCL3L3,IL4	3
	quantity of Ca2+	3.13E-03	CCL24,CCL3L1/CCL3L3,IL4	3
Skeletal and Muscular Disorders	rheumatoid arthritis	4.97E-03	CCL24,CCL3L1/CCL3L3,IL4	3
Skeletal and Muscular System Development and Function	formation of osteoclasts	2.44E-04	CCL3L1/CCL3L3,FGF18,IL4	3
Tissue Development	accumulation of leukocytes	1.61E-03	CCL24,CCL3L1/CCL3L3,IL4	3
	development of	1.75E-03	FGF18,HAND2,IL4	3

	connective tissue			
Tissue Morphology	quantity of granulocytes	1.98E-03	CCL24,CCL3L1/CCL3L3,IL4	3
	quantity of cells	4.84E-03	CCL24,CCL3L1/CCL3L3,FGF18,IL4	4
Vitamin and Mineral Metabolism	flux of Ca2+	1.75E-03	CCL24,CCL3L1/CCL3L3,IL4	3
	quantity of Ca2+	3.13E-03	CCL24,CCL3L1/CCL3L3,IL4	3

*IPA Top Molecular and Cellular Functions
