# Supplementary material for: Pubertal high fat diet: effects on mammary cancer development
Source: Breast Cancer Res. 2013 Oct 25;15(5):R100. doi: 10.1186/bcr3561 (PMC3978633; doi:10.1186/bcr3561)
Supplement: Additional file 8: Table S4 — Four weeks on diet qPCR Ingenuity Pathway Analysis. [file bcr3561-S8.pdf]

Supplemental Table 4. 4 weeks on diet qPCR Ingenuity Pathway Analysis

1) Significant Gene List (5):

BDNF
FZD5
LEFFTY2
LIF
TNFSF11/RANKL

2) Top Canonical Pathways

Ingenuity Canonical Pathways	B-H Adjusted p-value	Ratio	Molecules
**Human Embryonic Stem Cell Pluripotency**	**1.44E-04**	**3/156**	**BDNF,FZD5,LEFTY2**
**Mouse Embryonic Stem Cell Pluripotency**	**4.07E-03**	**2/99**	**LIF,FZD5**
**Role of NANOG in Mammalian Embryonic Stem Cell Pluripotency**	**4.07E-03**	**2/114**	**LIF,FZD5**
**Role of Osteoblasts, Osteoclasts and Chondrocytes in Rheumatoid Arthritis**	**1.20E-02**	**2/238**	**TNFSF11,FZD5**
**Role of Macrophages, Fibroblasts and Endothelial Cells in Rheumatoid Arthritis**	**1.83E-02**	**2/332**	**TNFSF11,FZD5**
Axonal Guidance Signaling	3.03E-02	2/469	BDNF,FZD5

3) Function Table

Category	Functions Annotation	B-H Adjusted p-value	Molecules	Number of Molecules
4wk qPCR IPA Function Table				
Cardiovascular System Development and Function	**development of cardiovascular system**	**4.06E-04**	**BDNF,FZD5,LEFTY2,LIF,TNFSF11**	**5**
	angiogenesis	1.19E-03	BDNF,FZD5,LIF,TNFSF11	4
	development of blood vessel	1.60E-03	BDNF,FZD5,LIF,TNFSF11	4
	proliferation of endothelial cells	1.86E-03	BDNF,FZD5,TNFSF11	3
	morphology of cardiovascular system	3.09E-03	FZD5,LEFTY2,LIF	3
Cell Death and Survival*	cell viability of tumor cell lines	3.36E-03	BDNF,LIF,TNFSF11	3
Cell Morphology*	size of connective tissue cells	4.66E-04	BDNF,LIF,TNFSF11	3
Cell Signaling	release of Ca2+	1.19E-03	BDNF,LIF,TNFSF11	3
Cell-mediated Immune Response	differentiation of T lymphocytes	2.03E-03	FZD5,LIF,TNFSF11	3
Cell-To-Cell Signaling and Interaction	stimulation of cells	1.86E-03	BDNF,LIF,TNFSF11	3
Cellular Development*	**maturation of cells**	**4.06E-04**	**BDNF,FZD5,LIF,TNFSF11**	**4**
	proliferation of embryonic	1.53E-03	BDNF,LIF,TNFSF11	3

	cells			
	proliferation of endothelial cells	1.86E-03	BDNF,FZD5,TNFSF11	3
	proliferation of muscle cells	2.03E-03	BDNF,LIF,TNFSF11	3
	differentiation of T lymphocytes	2.03E-03	FZD5,LIF,TNFSF11	3
	differentiation of tumor cell lines	2.03E-03	BDNF,LIF,TNFSF11	3
	differentiation of cells	4.29E-03	BDNF,FZD5,LIF,TNFSF11	4
Cellular Function and Maintenance*	**maintenance of cells**	**4.06E-04**	**BDNF,LIF,TNFSF11**	**3**
	differentiation of T lymphocytes	2.03E-03	FZD5,LIF,TNFSF11	3
Cellular Growth and Proliferation*	generation of cells	1.53E-03	BDNF,LIF,TNFSF11	3
	proliferation of embryonic cells	1.53E-03	BDNF,LIF,TNFSF11	3
	proliferation of endothelial cells	1.86E-03	BDNF,FZD5,TNFSF11	3
	stimulation of cells	1.86E-03	BDNF,LIF,TNFSF11	3
	proliferation of muscle cells	2.03E-03	BDNF,LIF,TNFSF11	3
	formation of cells	2.03E-03	BDNF,LIF,TNFSF11	3
Cellular Movement	chemotaxis of cells	2.47E-03	BDNF,LIF,TNFSF11	3
Connective Tissue Disorders	rheumatoid arthritis	3.47E-03	BDNF,FZD5,TNFSF11	3
Developmental Disorder	Growth Failure	3.00E-03	BDNF,LIF,TNFSF11	3
Digestive System Development and Function	morphology of digestive system	8.93E-04	BDNF,LEFTY2,LIF,TNFSF11	4
DNA Replication, Recombination, and Repair	synthesis of DNA	2.03E-03	BDNF,LIF,TNFSF11	3
Embryonic Development	proliferation of embryonic cells	1.53E-03	BDNF,LIF,TNFSF11	3
	development of body axis	3.00E-03	FZD5,LEFTY2,LIF	3
Gene Expression	transcription of RNA	3.83E-03	BDNF,FZD5,LIF,TNFSF11	4
Hematological System Development and Function	quantity of antigen presenting cells	1.58E-03	BDNF,LIF,TNFSF11	3
	differentiation of T lymphocytes	2.03E-03	FZD5,LIF,TNFSF11	3
Hematopoiesis	differentiation of T lymphocytes	2.03E-03	FZD5,LIF,TNFSF11	3
Immunological Disease	rheumatoid arthritis	3.47E-03	BDNF,FZD5,TNFSF11	3
Inflammatory Disease	rheumatoid arthritis	3.47E-03	BDNF,FZD5,TNFSF11	3
Lymphoid Tissue Structure and Development	differentiation of T lymphocytes	2.03E-03	FZD5,LIF,TNFSF11	3
	morphology of lymphatic system component	2.25E-03	LEFTY2,LIF,TNFSF11	3
Molecular Transport	release of Ca2+	1.19E-03	BDNF,LIF,TNFSF11	3
Organismal Development	development of blood vessel	1.60E-03	BDNF,FZD5,LIF,TNFSF11	4
	proliferation of endothelial cells	1.86E-03	BDNF,FZD5,TNFSF11	3
	development of body axis	3.00E-03	FZD5,LEFTY2,LIF	3
	size of body	5.13E-03	BDNF,LIF,TNFSF11	3

Organismal Injury and Abnormalities	Bleeding	2.03E-03	BDNF,FZD5,LIF	3
Organismal Survival	**organismal death**	**2.03E-03**	**BDNF,FZD5,LEFTY2,LIF,TNFSF11**	**5**
	survival of organism	3.00E-03	BDNF,FZD5,LIF	3
Skeletal and Muscular Disorders	rheumatoid arthritis	3.47E-03	BDNF,FZD5,TNFSF11	3
Skeletal and Muscular System Development and Function	proliferation of muscle cells	2.03E-03	BDNF,LIF,TNFSF11	3
	morphology of skeletal system	2.47E-03	LEFTY2,LIF,TNFSF11	3
Small Molecule Biochemistry	release of Ca2+	1.19E-03	BDNF,LIF,TNFSF11	3
Tissue Development	generation of cells	1.53E-03	BDNF,LIF,TNFSF11	3
	proliferation of endothelial cells	1.86E-03	BDNF,FZD5,TNFSF11	3
Tissue Morphology	quantity of antigen presenting cells	1.58E-03	BDNF,LIF,TNFSF11	3
Vitamin and Mineral Metabolism	release of Ca2+	1.19E-03	BDNF,LIF,TNFSF11	3

*IPA Top Molecular and Cellular Functions
