# Supplementary material for: Pubertal high fat diet: effects on mammary cancer development
Source: Breast Cancer Res. 2013 Oct 25;15(5):R100. doi: 10.1186/bcr3561 (PMC3978633; doi:10.1186/bcr3561)
Supplement: Additional file 9: Table S5 — Ten weeks on diet qPCR Ingenuity Pathway Analysis. [file bcr3561-S9.pdf]

Supplemental Table 5. 10 weeks on diet qPCR Ingenuity Pathway Analysis

1) Significant Gene List (24):

BMP2
BMP3
CCL1
CCL17
CCL19
CCL20
CCL22
CXCL12
CXCR2
FGF10
GDF10
GDF5
IL1A
IL1B
IL2
IL2RB
IL7
LTA
NODAL
PGF
TGFA
TGFB1
VEGFA
XCR1

2) Top Canonical Pathways

Ingenuity Canonical Pathways	B-H Adjusted p-value	Ratio	Molecules
**Granulocyte Adhesion and Diapedesis**	**5.66E-09**	**8/166**	**IL1A,CXCR2,CCL17,CCL20,IL1B,CCL22,CCL19, CCL1**
**Agranulocyte Adhesion and Diapedesis**	**5.66E-09**	**8/176**	**IL1A,CXCR2,CCL17,CCL20,IL1B,CCL22,CCL19, CCL1**
**Hepatic Fibrosis / Hepatic Stellate Cell Activation**	**1.55E-06**	**6/140**	**VEGFA,IL1A,TGFB1,TGFA,IL1B,PGF**
**Altered T Cell and B Cell Signaling in Rheumatoid Arthritis**	**3.24E-06**	**5/86**	**IL1A,IL2,TGFB1,LTA,IL1B**
**Role of Macrophages, Fibroblasts and Endothelial Cells in Rheumatoid Arthritis**	**3.32E-06**	**7/311**	**VEGFA,IL1A,TGFB1,LTA,IL1B,IL7,PGF**

Role of Osteoblasts, Osteoclasts and Chondrocytes in Rheumatoid Arthritis	1.12E-05	6/225	IL1A,TGFB1,BMP3,BMP2,IL1B,IL7
Role of Cytokines in Mediating Communication between Immune Cells	1.62E-05	4/52	IL1A,IL2,TGFB1,IL1B
NF-κB Signaling	5.11E-05	5/169	IL1A,LTA,BMP2,TGFA,IL1B
Factors Promoting Cardiogenesis in Vertebrates	1E-04	4/91	NODAL,TGFB1,BMP3,BMP2
Pancreatic Adenocarcinoma Signaling	1.98E-04	4/115	VEGFA,TGFB1,TGFA,PGF
Graft-versus-Host Disease Signaling	3.58E-04	3/46	IL1A,IL2,IL1B
Hematopoiesis from Pluripotent Stem Cells	3.58E-04	3/55	IL1A,IL2,IL7
Human Embryonic Stem Cell Pluripotency	3.61E-04	4/149	NODAL,TGFB1,BMP3,BMP2
Role of JAK1 and JAK3 in γc Cytokine Signaling	7.81E-04	3/63	IL2,IL7,IL2RB
Renal Cell Carcinoma Signaling	1.04E-03	3/71	VEGFA,TGFB1,TGFA
Hematopoiesis from Multipotent Stem Cells	1.04E-03	2/12	IL2,IL7
TGF-β Signaling	1.6E-03	3/89	NODAL,TGFB1,BMP2
Communication between Innate and Adaptive Immune Cells	1.6E-03	3/93	IL1A,IL2,IL1B

Bladder Cancer Signaling	1.68E-03	3/89	VEGFA,FGF10,PGF
Crosstalk between Dendritic Cells and Natural Killer Cells	1.76E-03	3/91	IL2,LTA,IL2RB
Differential Regulation of Cytokine Production in Intestinal Epithelial Cells by IL-17A and IL-17F	2.79E-03	2/23	IL1A,IL1B
Type I Diabetes Mellitus Signaling	2.79E-03	3/113	IL2,LTA,IL1B
Antiproliferative Role of TOB in T Cell Signaling	3.14E-03	2/26	IL2,TGFB1
IL-6 Signaling	3.14E-03	3/112	VEGFA,IL1A,IL1B
p38 MAPK Signaling	3.14E-03	3/116	IL1A,TGFB1,IL1B
Atherosclerosis Signaling	3.26E-03	3/131	IL1A,TGFB1,IL1B
Aryl Hydrocarbon Receptor Signaling	4.23E-03	3/141	IL1A,TGFB1,IL1B
Inhibition of Angiogenesis by TSP1	4.26E-03	2/32	VEGFA,TGFB1
Role of Hypercytokinemia/hyperchemokinemia in the Pathogenesis of Influenza	6.74E-03	2/41	IL1A,IL1B
RAR Activation	7.45E-03	3/175	VEGFA,TGFB1,BMP2
Dendritic Cell Maturation	7.45E-03	3/192	IL1A,LTA,IL1B
ILK Signaling	8.38E-03	3/185	VEGFA,BMP2,PGF

Clathrin-mediated Endocytosis Signaling	8.38E-03	3/191	VEGFA,FGF10,PGF
IL-8 Signaling	8.38E-03	3/192	VEGFA,CXCR2,PGF
IL-2 Signaling	9.28E-03	2/56	IL2,IL2RB
Axonal Guidance Signaling	1.05E-02	4/457	VEGFA,BMP3,BMP2,PGF
Systemic Lupus Erythematosus Signaling	1.12E-02	3/231	IL1A,IL2,IL1B
T Helper Cell Differentiation	1.35E-02	2/69	IL2,TGFB1
IL-10 Signaling	1.36E-02	2/72	IL1A,IL1B
Basal Cell Carcinoma Signaling	1.37E-02	2/72	BMP3,BMP2
Colorectal Cancer Metastasis Signaling	1.37E-02	3/244	VEGFA,TGFB1,PGF
BMP signaling pathway	1.49E-02	2/78	BMP3,BMP2
VEGF Family Ligand-Receptor Interactions	1.53E-02	2/78	VEGFA,PGF
Regulation of IL-2 Expression in Activated and Anergic T Lymphocytes	1.57E-02	2/84	IL2,TGFB1
Glucocorticoid Receptor Signaling	1.66E-02	3/277	IL2,TGFB1,IL1B
FXR/RXR Activation	1.74E-02	2/84	IL1A,IL1B
Role of Pattern Recognition Receptors in Recognition of Bacteria and Viruses	1.9E-02	2/95	IL2,IL1B
VEGF Signaling	1.9E-02	2/95	VEGFA,PGF
PPAR Signaling	1.9E-02	2/100	IL1A,IL1B

Telomerase Signaling	2.03E-02	2/99	IL2,IL2RB
Amyotrophic Lateral Sclerosis Signaling	2.03E-02	2/100	VEGFA,PGF
Nitric Oxide Signaling in the Cardiovascular System	2.03E-02	2/102	VEGFA,PGF
Cholecystokinin/Gastrin-mediated Signaling	2.11E-02	2/101	IL1A,IL1B
HIF1α Signaling	2.11E-02	2/102	VEGFA,PGF
iCOS-iCOSL Signaling in T Helper Cells	2.15E-02	2/112	IL2,IL2RB
Role of Tissue Factor in Cancer	2.27E-02	2/109	VEGFA,IL1B
Role of NANOG in Mammalian Embryonic Stem Cell Pluripotency	2.31E-02	2/113	BMP3,BMP2
Molecular Mechanisms of Cancer	2.62E-02	3/362	TGFB1,BMP3,BMP2
LXR/RXR Activation	2.68E-02	2/126	IL1A,IL1B
Gαi Signaling	2.68E-02	2/128	CXCR2,XCR1
eNOS Signaling	2.76E-02	2/129	VEGFA,PGF
Ovarian Cancer Signaling	3.02E-02	2/138	VEGFA,PGF
Hepatic Cholestasis	3.14E-02	2/141	IL1A,IL1B
PPARα/RXRα Activation	4.38E-02	2/173	TGFB1,IL1B
Acute Phase Response Signaling	4.51E-02	2/173	IL1A,IL1B
Ephrin Receptor Signaling	4.51E-02	2/197	VEGFA,PGF
Regulation of the Epithelial-Mesenchymal Transition Pathway	4.79E-02	2/187	FGF10,TGFB1

mTOR Signaling	4.87E-02	2/189	VEGFA,PGF

3) Function Table

Category	Functions Annotation	B-H Adjusted p-Value	Molecules	Number of Molecules
10wk qPCR IPA Function Tables				
**Cancer**	**Cancer**	**2.63E-03**	**BMP2,BMP3,CCL17,CCL19,CCL22,CXCR2,FGF10,GDF10,GDF5,IL1A,IL1B,IL2,IL2RB,IL7,LTA,PGF,TGFA,TGFB1,VEGFA,XCR1**	**20**
Cell Death and Survival	cell viability	8.92E-06	BMP2,IL1A,IL1B,IL2,IL2RB,IL7,LTA,TGFA,TGFB1,VEGFA	10
	apoptosis	2.43E-04	BMP2,FGF10,IL1A,IL1B,IL2,IL2RB,IL7,LTA,TGFA,TGFB1,VEGFA	11
	necrosis	2.56E-04	BMP2,IL1A,IL1B,IL2,IL2RB,IL7,LTA,PGF,TGFA,TGFB1,VEGFA	11
	cell death	2.83E-04	BMP2,FGF10,IL1A,IL1B,IL2,IL2RB,IL7,LTA,PGF,TGFA,TGFB1,VEGFA	12
**Cell-To-Cell Signaling and Interaction***	**signaling of cells**	**3.44E-14**	**BMP2,BMP3,CCL17,CCL20,CCL22,GDF5,IL1B,IL2,IL2RB,IL7,LTA,PGF**	**12**
	adhesion of immune cells	1.64E-13	CCL17,CCL19,CCL20,CCL22,CXCR2,IL1A,IL1B,IL2,IL7,TGFB1,VEGFA	11
	communication of cells	8.22E-13	BMP2,BMP3,CCL1,CCL17,CCL19,CCL20,CCL22,CXCR2,GDF5,IL1B,IL2,IL2RB,IL7,LTA,PGF,TGFB1,XCR1	17
	activation of cells	3.52E-09	BMP2,CXCR2,IL1A,IL1B,IL2,IL7,LTA,TGFA,TGFB1,VEGFA	10
	signal transduction	1.13E-05	CCL1,CCL17,CCL20,CCL22,CXCR2,IL1B,IL2RB,LTA,PGF,XCR1	10
Cellular Development*	differentiation of cells	5.04E-09	BMP2,BMP3,FGF10,IL1A,IL1B,IL2,IL2RB,IL7,PGF,TGFA,TGFB1,VEGFA	12
	proliferation of tumor cell lines	1.03E-06	BMP2,CCL20,CXCR2,FGF10,IL1A,IL1B,IL2,IL2RB,IL7,NODAL,TGFA,TGFB1,VEGFA	13
Cellular Function and Maintenance	cellular homeostasis	5.45E-07	CCL1,CCL19,CCL20,IL1B,IL2,IL2RB,IL7,TGFB1,VEGFA,XCR1	10
Cellular Growth and Proliferation*	proliferation of cells	3.08E-07	BMP2,CCL19,CCL20,CXCR2,FGF10,GDF5,IL1A,IL1B,IL2,IL2RB,IL7,LTA,NODAL,PGF,TGFA,TGFB1,VEGFA	17
	proliferation of tumor cell lines	1.03E-06	BMP2,CCL20,CXCR2,FGF10,IL1A,IL1B,IL2,IL2RB,IL7,NODAL,TGFA,TGFB1,VEGFA	13
Cellular Movement*	leukocyte migration	2.03E-13	CCL1,CCL17,CCL19,CCL20,CCL22,CXCR2,IL1A,IL1B,IL2,IL7,PGF,TGFB1,VEGFA	13
	migration of cells	8.22E-13	BMP2,CCL1,CCL17,CCL19,CCL20,CCL22,CXCR2,FGF10,IL1A,IL1B,IL2,IL7,LTA,PGF,TGFA,TGFB1,VEGFA	17
	cell movement of leukocytes	1.47E-12	CCL1,CCL17,CCL19,CCL20,CCL22,CXCR2,IL1A,IL1B,IL2,IL7,TGFB1,VEGFA	12
	chemotaxis of cells	1.47E-12	CCL1,CCL17,CCL19,CCL20,CCL22,CXCR2,FGF10,IL1B,IL2,PGF,TGFB1,VEGFA	12
	chemotaxis of leukocytes	3.21E-11	CCL1,CCL17,CCL19,CCL20,CCL22,CXCR2,IL1B,IL2,TGFB1,VEGFA	10

	cell movement of phagocytes	4.05E-11	CCL1,CCL19,CCL20,CCL22,CXCR2,IL1A,IL1B,IL2,TGFB1,VEGFA	10
	cell movement of tumor cell lines	9.18E-09	BMP2,CCL1,CCL17,CCL19,CCL20,CCL22,FGF10,IL1A,PGF,TGFA,TGFB1,VEGFA	12
Connective Tissue Disorders	arthritis	6.19E-11	BMP2,CCL1,CCL17,CCL19,CCL20,CXCR2,GDF5,IL1A,IL1B,IL2,IL2RB,IL7,LTA,TGFB1,VEGFA	15
	rheumatoid arthritis	1.68E-08	CCL1,CCL17,CCL19,CCL20,CXCR2,IL1B,IL2,IL2RB,IL7,LTA,TGFB1,VEGFA	12
Hematological System Development and Function	adhesion of immune cells	1.64E-13	CCL17,CCL19,CCL20,CCL22,CXCR2,IL1A,IL1B,IL2,IL7,TGFB1,VEGFA	11
	cell movement of leukocytes	1.47E-12	CCL1,CCL17,CCL19,CCL20,CCL22,CXCR2,IL1A,IL1B,IL2,IL7,TGFB1,VEGFA	12
	chemotaxis of leukocytes	3.21E-11	CCL1,CCL17,CCL19,CCL20,CCL22,CXCR2,IL1B,IL2,TGFB1,VEGFA	10
	cell movement of phagocytes	4.05E-11	CCL1,CCL19,CCL20,CCL22,CXCR2,IL1A,IL1B,IL2,TGFB1,VEGFA	10
Immune Cell Trafficking	adhesion of immune cells	1.64E-13	CCL17,CCL19,CCL20,CCL22,CXCR2,IL1A,IL1B,IL2,IL7,TGFB1,VEGFA	11
	leukocyte migration	2.03E-13	CCL1,CCL17,CCL19,CCL20,CCL22,CXCR2,IL1A,IL1B,IL2,IL7,PGF,TGFB1,VEGFA	13
	cell movement of leukocytes	1.47E-12	CCL1,CCL17,CCL19,CCL20,CCL22,CXCR2,IL1A,IL1B,IL2,IL7,TGFB1,VEGFA	12
	chemotaxis of leukocytes	3.21E-11	CCL1,CCL17,CCL19,CCL20,CCL22,CXCR2,IL1B,IL2,TGFB1,VEGFA	10
	cell movement of phagocytes	4.05E-11	CCL1,CCL19,CCL20,CCL22,CXCR2,IL1A,IL1B,IL2,TGFB1,VEGFA	10
Immunological Disease	rheumatoid arthritis	1.68E-08	CCL1,CCL17,CCL19,CCL20,CXCR2,IL1B,IL2,IL2RB,IL7,LTA,TGFB1,VEGFA	12
Inflammatory Disease	arthritis	6.19E-11	BMP2,CCL1,CCL17,CCL19,CCL20,CXCR2,GDF5,IL1A,IL1B,IL2,IL2RB,IL7,LTA,TGFB1,VEGFA	15
	rheumatoid arthritis	1.68E-08	CCL1,CCL17,CCL19,CCL20,CXCR2,IL1B,IL2,IL2RB,IL7,LTA,TGFB1,VEGFA	12
Inflammatory Response	inflammatory response	1.57E-12	CCL1,CCL17,CCL19,CCL20,CCL22,CXCR2,IL1A,IL1B,IL2,TGFB1,VEGFA,XCR1	12
	chemotaxis of leukocytes	3.21E-11	CCL1,CCL17,CCL19,CCL20,CCL22,CXCR2,IL1B,IL2,TGFB1,VEGFA	10
	cell movement of phagocytes	4.05E-11	CCL1,CCL19,CCL20,CCL22,CXCR2,IL1A,IL1B,IL2,TGFB1,VEGFA	10
Skeletal and Muscular Disorders	arthritis	6.19E-11	BMP2,CCL1,CCL17,CCL19,CCL20,CXCR2,GDF5,IL1A,IL1B,IL2,IL2RB,IL7,LTA,TGFB1,VEGFA	15
	rheumatoid arthritis	1.68E-08	CCL1,CCL17,CCL19,CCL20,CXCR2,IL1B,IL2,IL2RB,IL7,LTA,TGFB1,VEGFA	12
Tissue Development	adhesion of immune cells	1.64E-13	CCL17,CCL19,CCL20,CCL22,CXCR2,IL1A,IL1B,IL2,IL7,TGFB1,VEGFA	11

*IPA Top Molecular and Cellular Functions
