# Supplementary material for: Pubertal high fat diet: effects on mammary cancer development
Source: Breast Cancer Res. 2013 Oct 25;15(5):R100. doi: 10.1186/bcr3561 (PMC3978633; doi:10.1186/bcr3561)
Supplement: Additional file 10: Table S6 — Tumor qPCR Ingenuity Pathway Analysis. [file bcr3561-S10.pdf]

Supplemental Table 6. Tumor qPCR Ingenuity Pathway Analysis

1) Significant Gene List (15):

APAF1
BMP10
BMP7
BRCA1
BRCA2
CCND2
CTNNB1
IGF1R
INHA
KRT18
KRT8
NTF3
SLIT2
TERT
TP53/TRP53

2) Top Canonical Pathways

Ingenuity Canonical Pathways	B-H Adjusted p-value	Ratio	Molecules
**Molecular Mechanisms of Cancer**	**7.1E-07**	**7/378**	**TP53,CCND2,APAF1,BMP7,BRCA1,CTNNB1,BMP10**
**p53 Signaling**	**7.1E-07**	**5/96**	**TP53,CCND2,APAF1,BRCA1,CTNNB1**
**Basal Cell Carcinoma Signaling**	**1.27E-05**	**4/73**	**TP53,BMP7,CTNNB1,BMP10**
**GADD45 Signaling**	**1.43E-05**	**3/22**	**TP53,CCND2,BRCA1**
**Role of NANOG in Mammalian Embryonic Stem Cell Pluripotency**	**4.54E-05**	**4/114**	**TP53,BMP7,CTNNB1,BMP10**
Ovarian Cancer Signaling	7.16E-05	4/142	TP53,BRCA2,BRCA1,CTNNB1
Human Embryonic Stem Cell Pluripotency	7.16E-05	4/156	NTF3,BMP7,CTNNB1,BMP10
Thyroid Cancer Signaling	7.22E-05	3/42	TP53,NTF3,CTNNB1
Myc Mediated Apoptosis Signaling	1.98E-04	3/60	TP53,IGF1R,APAF1
Role of BRCA1 in DNA Damage Response	1.98E-04	3/65	TP53,BRCA2,BRCA1
Factors Promoting Cardiogenesis in Vertebrates	5.49E-04	3/94	BMP7,CTNNB1,BMP10
DNA Double-Strand Break Repair by Homologous Recombination	5.74E-04	2/17	BRCA2,BRCA1
Cardiomyocyte Differentiation via BMP Receptors	8.89E-04	2/20	BMP7,BMP10
DNA damage-induced 14-3-3σ Signaling	9.22E-04	2/21	TP53,BRCA1
Hereditary Breast Cancer Signaling	9.48E-04	3/128	TP53,BRCA2,BRCA1
Aryl Hydrocarbon Receptor Signaling	1.52E-03	3/161	TP53,CCND2,APAF1
Glioblastoma Multiforme Signaling	1.65E-03	3/164	TP53,IGF1R,CTNNB1
Axonal Guidance Signaling	2.61E-03	4/469	NTF3,BMP7,SLIT2,BMP10
Role of PKR in Interferon Induction and Antiviral Response	3.07E-03	2/46	TP53,APAF1
Cell Cycle: G2/M DNA Damage Checkpoint Regulation	3.37E-03	2/48	TP53,BRCA1
Role of Oct4 in Mammalian Embryonic Stem Cell Pluripotency	3.51E-03	2/45	TP53,BRCA1
Role of Osteoblasts, Osteoclasts and Chondrocytes in Rheumatoid Arthritis	4.03E-03	3/238	BMP7,CTNNB1,BMP10
Huntington's Disease Signaling	4.03E-03	3/240	TP53,IGF1R,APAF1
Endometrial Cancer Signaling	4.1E-03	2/57	TP53,CTNNB1
Role of CHK Proteins in Cell Cycle	4.4E-03	2/57	TP53,BRCA1

Checkpoint Control			
Induction of Apoptosis by HIV1	4.69E-03	2/65	TP53,APAF1
ATM Signaling	4.69E-03	2/61	TP53,BRCA1
Cell Cycle: G1/S Checkpoint Regulation	4.81E-03	2/66	TP53,CCND2
Estrogen-Dependent Breast Cancer Signaling	4.81E-03	2/72	TERT,IGF1R
Small Cell Lung Cancer Signaling	6.05E-03	2/89	TP53,APAF1
BMP signaling pathway	6.05E-03	2/80	BMP7,BMP10
Cyclins and Cell Cycle Regulation	7.04E-03	2/89	TP53,CCND2
Prostate Cancer Signaling	7.31E-03	2/98	TP53,CTNNB1
TGF-β Signaling	7.31E-03	2/89	INHA,BMP7
Apoptosis Signaling	7.97E-03	2/95	TP53,APAF1
Glioma Signaling	8.76E-03	2/112	TP53,IGF1R
Mouse Embryonic Stem Cell Pluripotency	8.76E-03	2/99	TP53,CTNNB1
Amyotrophic Lateral Sclerosis Signaling	9.06E-03	2/117	TP53,APAF1
Telomerase Signaling	9.19E-03	2/103	TP53,TERT
Pancreatic Adenocarcinoma Signaling	1.02E-02	2/120	TP53,BRCA2
PI3K/AKT Signaling	1.31E-02	2/144	TP53,CTNNB1
Wnt/β-catenin Signaling	2.39E-02	2/174	TP53,CTNNB1
ILK Signaling	2.74E-02	2/192	KRT18,CTNNB1
Colorectal Cancer Metastasis Signaling	4.22E-02	2/258	TP53,CTNNB1

3) Function Table

Category	Functions Annotation	B-H Adjusted p-value	Molecules	Number of Molecules
Tumor QPCR IPA Function Table				
Cancer	gonadal tumor	5.14E-07	BMP7,BRCA1,BRCA2,CCND2,CTNNB1,INHA,KRT8,SLIT2,TP53	9
	head and neck tumor	5.84E-07	BRCA1,BRCA2,CCND2,CTNNB1,IGF1R,KRT18,SLIT2,TERT,TP53	9
	hyperplasia	1.23E-06	APAF1,BRCA1,BRCA2,CCND2,CTNNB1,IGF1R,KRT8,TERT,TP53	9
	mammary tumor	1.61E-06	BMP7,BRCA1,BRCA2,CTNNB1,IGF1R,KRT18,KRT8,SLIT2,TERT,TP53	10
	genital tumor	3.11E-06	BMP7,BRCA1,BRCA2,CCND2,CTNNB1,IGF1R,INHA,KRT8,SLIT2,TP53	10
	gastrointestinal tract cancer	3.11E-06	BMP7,BRCA1,BRCA2,CCND2,CTNNB1,IGF1R,KRT18,SLIT2,TERT,TP53	10
	Ovarian Cancer and Tumors	3.11E-06	BMP7,BRCA1,BRCA2,CTNNB1,INHA,KRT8,SLIT2,TP53	8
	head and neck cancer	3.67E-06	BRCA1,BRCA2,CTNNB1,IGF1R,KRT18,SLIT2,TERT,TP53	8
	ovarian tumor	3.87E-06	BMP7,BRCA1,BRCA2,CTNNB1,INHA,KRT8,SLIT2,TP53	8
	breast cancer	9.34E-06	BMP7,BRCA1,BRCA2,CTNNB1,IGF1R,KRT8,SLIT2,TERT,TP53	9
	endocrine gland	1.31E-05	BRCA1,BRCA2,CCND2,CTNNB1,IGF1R,INHA,TP53	7

	tumor			
	ovarian cancer	2.73E-05	BMP7,BRCA1,BRCA2,CTNNB1,KRT8,SLIT2,TP53	7
	benign neoplasia	5.84E-05	CCND2,CTNNB1,IGF1R,INHA,SLIT2,TERT,TP53	7
	colorectal cancer	8.47E-05	BMP7,CCND2,CTNNB1,IGF1R,KRT18,SLIT2,TERT,TP53	8
	uterine cancer	2.09E-04	BRCA2,CTNNB1,IGF1R,KRT18,KRT8,TERT,TP53	7
Cardiovascular System Development and Function	development of cardiovascular system	8.37E-07	BMP10,BMP7,BRCA1,CCND2,CTNNB1,IGF1R,NTF3,SLIT2,TERT,TP53	10
Cell Cycle	**cell cycle progression**	**7.43E-10**	**APAF1,BMP7,BRCA1,BRCA2,CCND2,CTNNB1,IGF1R,INHA,KRT18,KRT8,NTF3,TERT,TP53**	**13**
	mitosis	5.30E-07	APAF1,BMP7,BRCA1,BRCA2,IGF1R,KRT18,NTF3,TP53	8
	interphase of tumor cell lines	1.35E-06	BMP7,BRCA1,CCND2,CTNNB1,IGF1R,TERT,TP53	7
	G1 phase	2.93E-06	BMP7,BRCA1,CCND2,CTNNB1,IGF1R,TERT,TP53	7
Cell Death and Survival	**apoptosis**	**1.61E-08**	**APAF1,BMP10,BMP7,BRCA1,BRCA2,CCND2,CTNNB1,IGF1R,INHA,KRT18,KRT8,NTF3,SLIT2,TERT,TP53**	**15**
	necrosis	2.01E-07	APAF1,BMP10,BMP7,BRCA1,BRCA2,CCND2,CTNNB1,IGF1R,INHA,KRT18,KRT8,NTF3,TERT,TP53	14
	cell death of connective tissue cells	4.13E-07	APAF1,BRCA1,BRCA2,CTNNB1,IGF1R,KRT18,KRT8,TERT,TP53	9
	apoptosis of muscle cells	4.13E-07	BMP10,BMP7,BRCA2,CTNNB1,IGF1R,TERT,TP53	7
	cell death of epithelial cells	4.41E-07	APAF1,BMP7,CTNNB1,IGF1R,KRT18,KRT8,TERT,TP53	8
	cell death of fibroblast cell lines	5.14E-07	APAF1,BRCA1,BRCA2,CTNNB1,KRT18,KRT8,TERT,TP53	8
	apoptosis of tumor cell lines	1.92E-06	APAF1,BMP7,BRCA1,BRCA2,CTNNB1,IGF1R,KRT18,NTF3,TERT,TP53	10
	cell viability	1.98E-05	APAF1,BMP7,BRCA1,BRCA2,CTNNB1,IGF1R,NTF3,TERT,TP53	9
	cell viability of tumor cell lines	4.98E-05	BRCA1,BRCA2,CTNNB1,IGF1R,NTF3,TERT,TP53	7
Cell Morphology	morphology of cells	2.01E-07	APAF1,BMP7,BRCA1,CCND2,CTNNB1,IGF1R,INHA,KRT18,KRT8,NTF3,TERT,TP53	12
	abnormal morphology of cells	7.69E-06	APAF1,BMP7,BRCA1,CCND2,IGF1R,INHA,KRT8,NTF3,TP53	9
Cellular Assembly and Organization	microtubule dynamics	3.56E-04	BMP7,CTNNB1,IGF1R,KRT18,NTF3,SLIT2,TP53	7
Cellular Development	proliferation of breast cancer cell lines	2.34E-06	BMP7,BRCA1,BRCA2,CCND2,IGF1R,TERT,TP53	7
	proliferation of tumor cell lines	7.85E-06	APAF1,BMP7,BRCA1,BRCA2,CCND2,CTNNB1,IGF1R,INHA,TERT,TP53	10
	differentiation of cells	1.19E-05	APAF1,BMP7,BRCA1,BRCA2,CTNNB1,IGF1R,INHA,KRT8,NTF3,TERT,TP53	11
Cellular Function and Maintenance	cellular homeostasis	2.80E-04	APAF1,BRCA1,CTNNB1,IGF1R,KRT18,KRT8,NTF3,TP53	8
	microtubule dynamics	3.56E-04	BMP7,CTNNB1,IGF1R,KRT18,NTF3,SLIT2,TP53	7
Cellular Growth	proliferation of breast	2.34E-06	BMP7,BRCA1,BRCA2,CCND2,IGF1R,TERT,TP53	7

and Proliferation	cancer cell lines			
	proliferation of epithelial cells	2.58E-06	BRCA1,CCND2,CTNNB1,IGF1R,INHA,TERT,TP53	7
	proliferation of cells	3.18E-06	APAF1,BMP10,BMP7,BRCA1,BRCA2,CCND2,CTNNB1,IGF1R,INHA,KRT8,NTF3,SLIT2,TERT,TP53	14
	proliferation of tumor cell lines	7.85E-06	APAF1,BMP7,BRCA1,BRCA2,CCND2,CTNNB1,IGF1R,INHA,TERT,TP53	10
Cellular Movement	migration of cells	3.78E-05	APAF1,BMP10,BMP7,BRCA1,CTNNB1,IGF1R,KRT8,NTF3,SLIT2,TP53	10
Developmental Disorder	Hypertrophy	5.14E-07	APAF1,BMP10,BMP7,CTNNB1,INHA,NTF3,TERT,TP53	8
	Hypoplasia	9.30E-06	BMP10,BMP7,BRCA2,CCND2,IGF1R,NTF3,TP53	7
Digestive System Development and Function	morphology of digestive system	1.96E-05	APAF1,BMP7,BRCA1,INHA,KRT18,KRT8,TP53	7
DNA Replication, Recombination, and Repair	synthesis of DNA	1.22E-09	BMP7,BRCA1,BRCA2,CCND2,CTNNB1,IGF1R,INHA,NTF3,TERT,TP53	10
Embryonic Development	development of brain	2.12E-09	APAF1,BMP7,BRCA2,CCND2,CTNNB1,IGF1R,NTF3,SLIT2,TERT,TP53	10
	development of genital organ	1.06E-05	BMP7,BRCA1,BRCA2,CCND2,CTNNB1,INHA,TP53	7
Endocrine System Disorders	Ovarian Cancer and Tumors	3.11E-06	BMP7,BRCA1,BRCA2,CTNNB1,INHA,KRT8,SLIT2,TP53	8
	ovarian cancer	2.73E-05	BMP7,BRCA1,BRCA2,CTNNB1,KRT8,SLIT2,TP53	7
Gastrointestinal Disease	gastrointestinal tract cancer	3.11E-06	BMP7,BRCA1,BRCA2,CCND2,CTNNB1,IGF1R,KRT18,SLIT2,TERT,TP53	10
	colorectal cancer	8.47E-05	BMP7,CCND2,CTNNB1,IGF1R,KRT18,SLIT2,TERT,TP53	8
Gene Expression	transactivation	2.56E-05	BMP7,BRCA1,BRCA2,CCND2,CTNNB1,TERT,TP53	7
Nervous System Development and Function	development of brain	2.12E-09	APAF1,BMP7,BRCA2,CCND2,CTNNB1,IGF1R,NTF3,SLIT2,TERT,TP53	10
	morphology of nervous system	1.15E-04	APAF1,BRCA1,CCND2,CTNNB1,IGF1R,NTF3,TP53	7
Organ Development	development of brain	2.12E-09	APAF1,BMP7,BRCA2,CCND2,CTNNB1,IGF1R,NTF3,SLIT2,TERT,TP53	10
	development of genital organ	1.06E-05	BMP7,BRCA1,BRCA2,CCND2,CTNNB1,INHA,TP53	7
Organismal Development	development of brain	2.12E-09	APAF1,BMP7,BRCA2,CCND2,CTNNB1,IGF1R,NTF3,SLIT2,TERT,TP53	10
	development of genital organ	1.06E-05	BMP7,BRCA1,BRCA2,CCND2,CTNNB1,INHA,TP53	7
	size of body	2.48E-05	BMP7,BRCA1,BRCA2,IGF1R,INHA,KRT8,NTF3,TP53	8
Organismal Survival	organismal death	1.87E-07	APAF1,BMP10,BMP7,BRCA1,BRCA2,CCND2,CTNNB1,IGF1R,INHA,KRT8,NTF3,SLIT2,TERT,TP53	14
	survival of organism	4.66E-07	BMP7,BRCA2,CCND2,CTNNB1,IGF1R,INHA,KRT18,NTF3,TP53	9
	perinatal death	3.42E-05	APAF1,BMP7,BRCA1,IGF1R,NTF3,SLIT2,TP53	7
Reproductive System Development	morphology of reproductive system	1.29E-06	APAF1,BMP7,BRCA1,BRCA2,IGF1R,INHA,KRT8,TP53	8
	development of	2.51E-06	BMP7,BRCA1,BRCA2,CCND2,CTNNB1,IGF1R,INHA,	8

and Function	reproductive system		TP53	
	development of genital organ	1.06E-05	BMP7,BRCA1,BRCA2,CCND2,CTNNB1,INHA,TP53	7
Reproductive System Disease	gonadal tumor	5.14E-07	BMP7,BRCA1,BRCA2,CCND2,CTNNB1,INHA,KRT8, SLIT2,TP53	9
	infertility	1.54E-06	APAF1,BMP7,BRCA2,CCND2,INHA,KRT8,TP53	7
	genital tumor	3.11E-06	BMP7,BRCA1,BRCA2,CCND2,CTNNB1,IGF1R,INHA, KRT8,SLIT2,TP53	10
	Ovarian Cancer and Tumors	3.11E-06	BMP7,BRCA1,BRCA2,CTNNB1,INHA,KRT8,SLIT2,T P53	8
	ovarian tumor	3.87E-06	BMP7,BRCA1,BRCA2,CTNNB1,INHA,KRT8,SLIT2,T P53	8
	ovarian cancer	2.73E-05	BMP7,BRCA1,BRCA2,CTNNB1,KRT8,SLIT2,TP53	7
	uterine cancer	2.09E-04	BRCA2,CTNNB1,IGF1R,KRT18,KRT8,TERT,TP53	7
Tissue Development	development of brain	2.12E-09	APAF1,BMP7,BRCA2,CCND2,CTNNB1,IGF1R,NTF3, SLIT2,TERT,TP53	10
	development of genital organ	1.06E-05	BMP7,BRCA1,BRCA2,CCND2,CTNNB1,INHA,TP53	7
Tissue Morphology	morphology of embryonic tissue	2.36E-08	APAF1,BMP10,BMP7,BRCA1,BRCA2,CTNNB1,KRT8 ,NTF3,SLIT2,TP53	10
	abnormal morphology of embryonic tissue	2.48E-07	APAF1,BMP7,BRCA1,BRCA2,CTNNB1,KRT8,NTF3, SLIT2,TP53	9
	quantity of cells	5.30E-07	APAF1,BMP7,BRCA1,BRCA2,CCND2,CTNNB1,IGF1 R,INHA,KRT8,NTF3,TERT,TP53	12
