# Supplementary material for: Pubertal high fat diet: effects on mammary cancer development
Source: Breast Cancer Res. 2013 Oct 25;15(5):R100. doi: 10.1186/bcr3561 (PMC3978633; doi:10.1186/bcr3561)
Supplement: Additional file 11: Figure S5 — Correlations between cytokine mRNA expression and leukocyte recruitment. Cycle threshold (Ct) values for the indicated mRNA in individual animals were normalized to the average Ct values for GusB, Hprt1, Hsp90ab1, Gapdh, and Actb mRNA in the same animals, and plotted against the indicated leukocyte counts in the same animals in association with the indicated individual structures. Correlations (r) were calculated by Spearman’s method. [file bcr3561-S11.pdf]

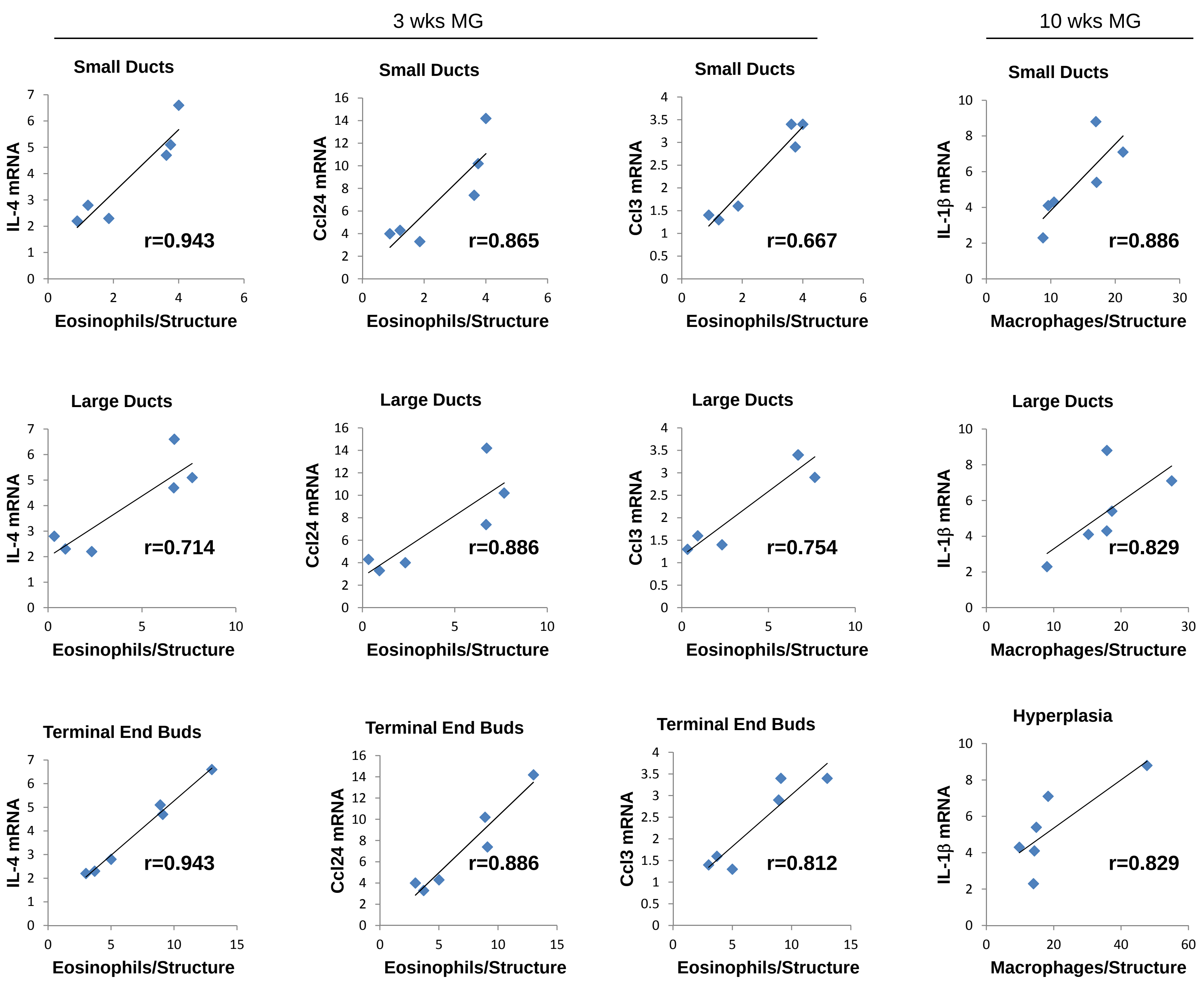

Figure S5. Correlations between cytokine mRNA expression and leukocyte recruitment. Ct values for the indicated mRNAs in individual animals were normalized to the average Ct values for *GusB*, *Hprt1*, *Hsp90ab1*, *Gapdh*, and *Actb* mRNAs in the same animals, and plotted against the indicated leukocyte counts in the same animals in association with the indicated individual structures. Correlations (r) were calculated by Spearman's method.
